# Using Zeolite Materials to Remove Pharmaceuticals from Water

**DOI:** 10.3390/ma17153848

**Published:** 2024-08-03

**Authors:** Tomasz Bajda, Agnieszka Grela, Justyna Pamuła, Joanna Kuc, Agnieszka Klimek, Jakub Matusik, Wojciech Franus, Santhana Krishna Kumar Alagarsamy, Tomasz Danek, Paweł Gara

**Affiliations:** 1 Faculty of Geology, Geophysics and Environmental Protection, AGH University of Krakow, al. A. Mickiewicza 30, 30-059 Krakow, Poland; aklimek@agh.edu.pl (A.K.); jmatusik@agh.edu.pl (J.M.); tdanek@agh.edu.pl (T.D.); 2 Faculty of Environmental and Power Engineering, Cracow University of Technology, ul. Warszawska 24, 31-155 Krakow, Poland; agnieszka.grela@pk.edu.pl (A.G.); justyna.pamula@pk.edu.pl (J.P.); 3 Faculty of Chemical Engineering and Technology, Cracow University of Technology, ul. Warszawska 24, 31-155 Krakow, Poland; joanna.kuc@pk.edu.pl; 4 Faculty of Civil Engineering and Architecture, Lublin University of Technology, ul. Nadbystrzycka 40, 20-618 Lublin, Poland; w.franus@pollub.pl; 5 Department of Chemistry, National Sun Yat-sen University, 70 Lienhai Road, Kaohsiung 80424, Taiwan; krishnakumar@mail.nsysu.edu.tw; 6 Faculty of Mechanical Engineering and Robotics, AGH University of Krakow, al. A. Mickiewicza 30, 30-059 Krakow, Poland; pgara@agh.edu.pl

**Keywords:** adsorption, zeolite, carbon–zeolite composite, wastewater, antibiotics

## Abstract

Pharmaceutical drugs, including antibiotics and hormonal agents, pose a significant threat to environmental and public health due to their persistent presence in aquatic environments. Colistin (KOL), fluoxetine (FLUO), amoxicillin (AMO), and 17-alpha-ethinylestradiol (EST) are pharmaceuticals (PhCs) that frequently exceed regulatory limits in water and wastewater. Current removal methods are mainly ineffective, necessitating the development of more efficient techniques. This study investigates the use of synthetic zeolite (NaP1_FA) and zeolite-carbon composites (NaP1_C), both derived from fly ash (FA), for the removal of KOL, FLUO, AMO, and EST from aquatic environments. Batch adsorption experiments assessed the effects of contact time, adsorbent dosage, initial concentration, and pH on the removal efficiency of the pharmaceuticals. The results demonstrated that NaP1_FA and NaP1_C exhibited high removal efficiencies for all tested pharmaceuticals, achieving over 90% removal within 2 min of contact time. The Behnajady-Modirshahla-Ghanbary (BMG) kinetic model best described the adsorption processes. The most effective sorption was observed with a sorbent dose of 1–2 g L^−1^. Regarding removal efficiency, the substances ranked in this order: EST was the highest, followed by AMO, KOL, and FLUO. Sorption efficiency was influenced by the initial pH of the solutions, with optimal performance observed at pH 2–2.5 for KOL and FLUO. The zeolite-carbon composite NaP1_C, due to its hydrophobic nature, showed superior sorption efficiency for hydrophobic pharmaceuticals like FLUO and EST. The spectral analysis reveals that the primary mechanism for immobilizing the tested PhCs on zeolite sorbents is mainly due to physical sorption. This study underscores the potential of utilizing inexpensive, fly ash-derived zeolites and zeolite-carbon composites to remove pharmaceuticals from water effectively. These findings contribute to developing advanced materials for decentralized wastewater treatment systems, directly addressing pollution sources in various facilities.

## 1. Introduction

Emerging pollutants, such as pharmaceutical drugs and their metabolites, pose a grave threat to the environment and public health. These compounds find their way into surface and groundwater, jeopardizing drinking water quality and disrupting aquatic life’s activities and functions. Moreover, they play a role in the proliferation of drug-resistant pathogens [1]. Unfortunately, existing wastewater treatment systems are ill-equipped to remove these pharmaceutical contaminants effectively [2,3,4]. Municipal wastewater treatment plants handle an array of wastewater sources, including domestic wastewater from homes, stores, and offices, industrial waste from chemical and other factories, agricultural runoff from livestock farms, medical waste from hospitals and clinics, pharmaceutical waste from manufacturing facilities, as well as waste from laboratories and even rainwater. In these collective treatment systems, dealing with such a diverse mixture of pollutants poses a significant challenge. Specifically, disposing of pharmaceuticals is particularly difficult. Their concentrations in the wastewater are too low to be effectively targeted by the microorganisms used in biological treatment processes. Furthermore, chemical methods struggle with this complex pollutant mix, as they lack selectivity and can have numerous side effects. In addition, various chemical reactions during wastewater treatment can produce metabolites and transformation products of pharmaceuticals (PhCs), making the treated wastewater a source of surface water pollution [1,5,6]. At the same time, pharmaceuticals are arousing concern and interest among scientists and the general public [7]. The reason is that these compounds negatively affect fish and freshwater invertebrates and contribute to the development of antibiotic-resistant bacteria [8,9,10].

The quest for advanced materials and technologies to remove PhCs from wastewater is critical. Techniques like adsorption or photocatalytic degradation are known to eliminate PhCs and their byproducts [11]. Our innovative research focuses on creating mineral and mineral-organic composites that function as sorbents. We advocate using novel, cost-effective sorbents, such as zeolites and zeolite-carbon composites produced from fly ash. Zeolites are particularly effective, as they can sorb pollutants at very low concentrations, can be regenerated and reused, and are inexpensive to produce [12]. The unique architecture of the zeolites group consists of intricate three-dimensional aluminum and silicon tetrahedron frameworks, forming water-filled channels and interchangeable alkali or alkaline earth metal cations. These distinctive structural characteristics make zeolites highly valuable in the separation and refining industries, where they serve as essential catalysts, adsorbents, and ion exchangers [13]. Synthetic zeolites (NaP1_FA) and zeolite-carbon composites (NaP1_C) were used as sorbents.

The present work reports the results of adsorption efficiencies from solutions through selected PhCs: KOL, FLUO, AMO, and EST. KOL and AMO are different-generation antibiotics, while FLUO is an antidepressant, and EST is a hormonal drug. KOL is an antibiotic used against Gram-negative bacteria in humans [14]. Globally, it is used as a veterinary drug to prevent and control disease in livestock and poultry [15,16]. Such intensive use of KOL in livestock farming results in the introduction of large quantities of this antibiotic into the environment. This potentially creates a risk of colistin-resistant bacteria and thus contributes to negative environmental impacts [17,18]. FLUO (known as Prozac^®^, Eli Lilly and Company, Indianapolis, IN, USA) is one of the most commonly prescribed antidepressants [19,20]. FLUO has been detected in raw and treated wastewater [21,22,23], in surface water [24,25], and even in drinking water [26]. AMO is a broad-spectrum β-lactam antibiotic. It is used in veterinary treatment (gastrointestinal and systemic infections). Due to its broad spectrum of action against bacteria, it is widely used in humans [27]. AMO is difficult to degrade. Removing amoxicillin from the environment is considered necessary, although it is associated with high costs. Among the methods available for this purpose is the adsorption process [28]. However, few research results are still available on removing antibiotics from wastewater using alternative adsorbents [29,30]. EST is a synthetic hormone found in oral contraceptives [31] and is a hydrophobic pharmaceutical [32]. In 2018, EST was added to the European Watch List [33] because its widespread use in human medicine and animal husbandry has increased environmental occurrence. Studies have shown that EST is highly resistant to oxidation in the environment due to the ethyl group at position 17 due to its high carbon-carbon bond energy [34]. EST is recognized as one of the main contributors to the estrogenic effects of wastewater [35]. Even though the levels of EST in wastewater are quite low, typically just a few nanograms per liter, its powerful effects can still induce hormonal changes in animals [36]. To justify the selection of KOL, FLUO, AMO, and EST, we present their physicochemical properties in Table 1 [37,38].

The purpose of the ongoing research was to test the applicability of zeolite (NaP1_FA) and zeolite-carbon composite (NaP1_C) as potential adsorbents for the removal of KOL, FLUO, AMO, and EST from aqueous solutions. Fly ash was used in the synthesis process to obtain the adsorbents, which can be considered a possibility for reuse [39]. This makes these adsorbents inexpensive materials that can be used to purify aqueous solutions from PhCs. In the paper [40], the authors presented a detailed characterization of the adsorbents studied using SEM, N_2_ adsorption/desorption, XRD, FTIR spectroscopy, and STA/TG analysis. This work focuses on the results of sorption experiments. The novelty of this work is to analyze the effectiveness of using two different fly ash-based adsorbents to determine their ability to remove KOL, FLUO, AMO, and EST from aqueous solutions.

The four selected PhCs possess diverse physicochemical properties, including varying levels of polarity, solubility, acid dissociation constants, partition coefficients, and stability in both acidic and basic environments. This diversity makes their simultaneous determination challenging. Despite extensive research on multi-residue analysis of pharmaceuticals, a single chromatographic method for their concurrent determination is absent from the literature. For instance, being hydrophilic and polar, KOL is more effectively detected using a hydrophilic column. In contrast, FLUO, which is lipophilic and moderately polar, is better separated with a reversed-phase C18 column [41,42]. In our research, we devised an HPLC-MS method tailored for the simultaneous detection of KOL and FLUO. This method provides exceptional selectivity across an extensive elution window while enhancing retention for both polar and non-polar analytes. 

The authors are confident that the experimental studies will add to the knowledge needed for real-scale research.

## 2. Materials and Methods

### 2.1. Materials and Chemicals

Zeolite NaP1_FA was synthesized from FA using an established method [43]. This involved a 24-h hydrothermal synthesis at 353 K, where FA was reacted with NaOH under atmospheric pressure. The zeolite–carbon NaP1_C composite followed a different procedure [44]. In this method, 200 g of carbon-rich FA (HiC) was mixed with 1000 mL of a 3 M NaOH solution and stirred at 80 °C for 48 h. The mixture was rinsed six times with 500 mL of distilled water and then dried at 105 °C. The primary difference between NaP1_FA and NaP1_C lies in the carbon content of the FA used. NaP1_FA utilized F-class FA from the Rybnik Power Plant in Rybnik, Poland, which contains less than 5% unburned carbon [39]. Conversely, NaP1_C was synthesized using high-carbon FA from the Janikowo Thermal Power Station (Inowrocław, Poland) [44], containing approximately 40% unburned carbon. Consequently, the resulting carbon contents differed significantly, with NaP1_FA at 5.53% and NaP1_C at 42.19%. The detailed mineral composition, chemical composition, and physicochemical properties of NaP1_FA and NaP1_C are presented in the work of [40]. 

KOL, FLUO, AMO, EST, acetonitrile LC-MS Optima^®^ (Optima Chemical, Douglas, AZ, USA), and formic acid LC-MS Optima^®^ were purchased from Sigma-Aldrich. All reagents were of analytical grade. To prepare stock solutions with a concentration of 1.0 µg mL^−1^, PhCs were dissolved meticulously in acetonitrile. Solutions (with concentrations > 1 mg L^−1^) for the experiments of the effect of the initial concentrations were prepared by dissolving the reagents at 60 °C and simultaneously treating them with ultrasound. The prepared solutions were kept in a refrigerator and stored for six months. Before analysis, the solutions were passed through a 0.22-µm syringe filter equipped with a nylon membrane. The physical and chemical properties of the PhCs analyzed are summarized in Table 1.

### 2.2. Adsorption and Desorption Experiments

The batch experiments employed two types of adsorbents, NaP1_FA and NaP1_C. Unless specified otherwise, all tests were conducted in duplicates, maintaining a solid/solution ratio of 1.0 g L^−1^. After a reaction period of 1 h, the suspensions underwent centrifugation at 4500 rpm for 10 min, followed by filtration using a 0.22 μm syringe filter before HPLC analysis. Moreover, before initiating reactions with the PhCs, all solid adsorbents were preconditioned by shaking them in water for an hour. Equilibrium pH was measured in every instance. 

Initially, PhCs adsorption experiments were carried out with a concentration of 100 μg L^−1^ to examine the adsorbents’ removal efficiency. Complete adsorption isotherms were determined within a 0.1–200 mg L^−1^ concentration range. For desorption, samples from these experiments were treated with 20 mL of ethanol. Furthermore, for samples reacted with 100 μg L^−1^ PhCs concentration, five successive cycles of regeneration using ethanol were performed. The effect of pH was analyzed at an initial PhCs concentration of 100 μg L^−1^, with starting pH values of 2.0, 2.5, and 8.0. The dosage effect was explored using adsorbent doses of 0.1, 0.2, 0.5, 1.0, and 2.0 g L^−1^. Lastly, PhCs’ adsorption kinetics were assessed for an initial concentration of 100 μg L^−1^ over contact times ranging from 0.5 to 360 min.

To test the reusability of NaP1_FA and NaP1_C adsorbents in terms of KOL, FLUO, AMO, and EST adsorption, five regeneration cycles (adsorption/desorption tests) were carried out. The adsorption cycles were carried out under similar conditions as described above using an adsorbent dosage of 1.0 g L^−1^ and an adsorbate concentration of 100 μg L^−1^. Following each adsorption cycle, the samples underwent centrifugation. The resulting solution was then gathered and prepared for HPLC analysis. After adsorption, 20 mL of ethanol was added to the adsorbent samples and shaken for 1 h to desorb KOL, FLUO, AMO, and EST. The samples underwent centrifugation, followed by thorough washing with distilled water, and were subsequently dried in preparation for the next adsorption cycle.

NaP1_FA and NaP1_C adsorbents were conditioned by adding 10 mL of redistilled water and shaking for 1 h. 

Using a Thermo Scientific Nicolet 6700 spectrometer (Thermo Fisher Scientific, Waltham, MA, USA) in transmission mode, FTIR spectroscopy analyses of sorbents were conducted before and after the sorption of PhCs (from an initial concentration of 200 mg L^−1^). The FTIR spectra were obtained from standard KBr pellets, containing 1 wt% sample/KBr, across the range of 4000–400 cm⁻^1^ (64 scans at a resolution of 4 cm⁻^1^).

### 2.3. Adsorption and Desorption Experiments

Determination of KOL, FLUO, AMO I EST was performed using Modular (U) HPLC system—Nexera (LC-40, Framingham, MA, USA) series, Shimadzu with a triple quadrupole analyzer and a QTrap 3200 reaction chamber (AB Sciex). Electrospray ionization (ESI) and the mode of multiple reaction monitoring (MRM) were applied. A KOL, FLUO, AMO, and EST standard solution with a concentration of 1 μg mL^−1^ was used to optimize the ionization parameters and detection. Once the precursor ion was chosen, the collision energy was fine-tuned to determine the optimal fragmentation pathway, ensuring the highest MS/MS response signal for the resulting product ions. 

The chromatographic separation was performed in an isocratic system on a Kinetex 2.6 μm XB C18 100A, 100 × 2.1 mm column with a SecurityGuard Ultra cartridges C18 precolumn (both from Phenomenex). The mobile phase was a mixture of (i) 0.2% formic acid in water LC-MS and (ii) 0.2% formic acid in acetonitrile LC-MS.

## 3. Results and Discussion

### 3.1. The Effect of the Initial Concentrations

**Figure 1:**
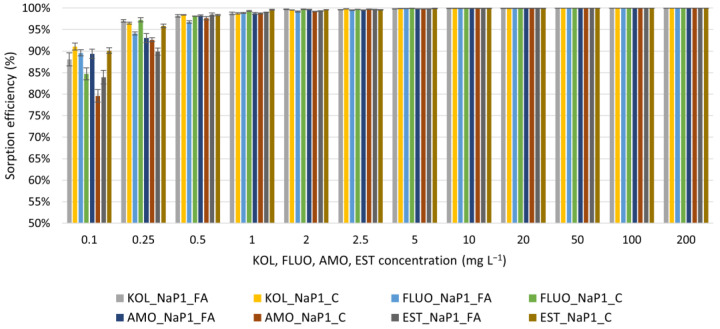
Effect of KOL, FLUO, AMO, and EST starting concentrations on their sorption efficiency on NaP1_FA and NaP1_C ([47], changed). An adsorbent dosage of 1.0 g L^−1^.

NaP1_FA and NaP1_C obtain high removal efficiencies of KOL, FLUO, AMO, and EST from solutions with different initial concentrations, ranging from 0.1 to 200 mg L^−1^ (Figure 1). The primary reason for the variation in PhCs sorption efficiency is their molecular size and structure [45,46]. Smaller molecules or those with a more linear structure may diffuse more easily into the pores of NaP1_FA and NaP1_C than larger or more complex molecules. EST and FLUO are slightly smaller molecules than AMO and noticeably smaller than KOL (Table 1). Also, the structure of these three PhCs is more linear than KOL. This would suggest EST, FLUO, and AMO should be absorbed with markedly higher efficiency than KOL. However, the results obtained (Figure 1) do not indicate such a tendency, especially in the range of the lowest concentration used (100 µg L^−1^). The second important parameter affecting the sorption efficiency of PhCs is hydrophobicity, which should positively correlate with the sorption efficiency of NaP1_C composite containing 42% carbon [40], whose surface is hydrophobic. In contrast, the dominance of the NaP1 zeolite in the NaP1_FA sample should be inversely correlated with the sorption efficiency of PhCs. To determine the hydrophobicity of the compounds, we can refer to their octanol-water partition coefficient (log P) values, which indicate how hydrophobic a substance is. The hydrophobicity of the investigated PhCs decreases in order FLUO > EST > AMO > KOL (Table 1). Besides, pharmaceuticals with low solubility in water may be more readily removed by sorbents due to the lower competition with water molecules for sorption sites. FLUO and EST have significantly lower water solubility than the other PhCs (Table 1). Considering the molecules’ size, the structure’s complexity, hydrophobicity, and water solubility of PhCs, one would expect EST and FLUO to sorb in more significant amounts than AMO and KOL. The results shown in Figure 1 generally confirm this thesis for the NaP1_C sample for initial concentrations of 0.1–0.5 mg L^−1^. For higher initial concentrations, the differences between the removal efficiencies of the individual PhCs from the solution blur.

Despite being a very large molecule with high efficiency and having the lowest logP value of all PhCs tested, KOL sorbs with high efficiency. The reason is the polarity and the presence of active groups on the surface of the KOL molecule. Pharmaceuticals with polar functional groups may interact more strongly with the polar sites on zeolites. KOL has polar amine and hydroxyl groups that might form hydrogen bonds with the zeolite surface, enhancing sorption efficiency.

**Figure 2:**
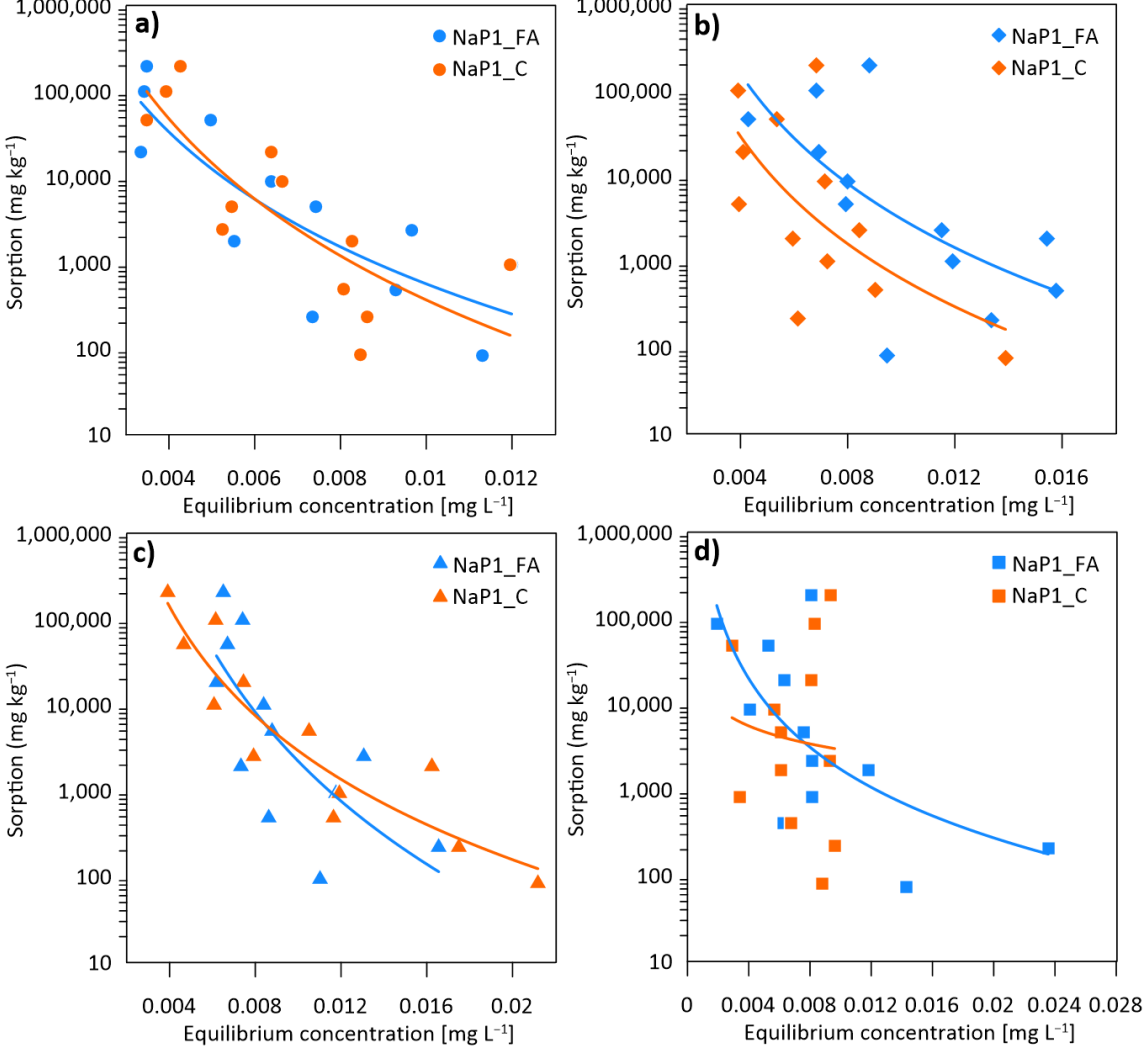
The adsorption isotherms of (**a**) KOL, (**b**) FLUO, (**c**) AMO, and (**d**) EST on NaP1_FA and NaP1_C.

Figure 2 demonstrates the changes in adsorption capacities relative to the equilibrium concentrations of PhCs. Higher equilibrium concentrations correspond to lower initial concentrations of PhCs when the percent sorption efficiency was lower (Figure 1). As the initial concentration increased, lower equilibrium concentrations and, at the same time, higher sorption were observed, corresponding to the highest (close to 100%) percent sorption efficiency (Figure 1). The resulting curves suggest that the adsorption process is complex and poorly understood. To address this, we tested theoretical models that provide experimental approaches to uncover the actual mechanism behind adsorption. The research data were analyzed by fitting them into the Langmuir, Freundlich, Dubinin-Radushkevich, and Temkin isotherm models to determine the most suitable model for design considerations. Details of models and results are presented in Supplementary Material (Text S1) in Figures S1–S16 and Table S1. None of the modeling results obtained sufficiently describe the sorption mechanisms. None of the tested models explains the course of the obtained isotherms, where sorption increases as the equilibrium concentration decreases. This behavior can occur due to various reasons. In studies of moisture sorption, the equilibrium moisture content can decrease as relative humidity increases [48], which is somewhat analogous to our observations. This phenomenon can occur due to changes in the material properties or competitive sorption at different sites. In some adsorption studies, it is observed that the adsorption capacity increases with increasing initial concentration due to the higher driving force for mass transfer [49]. The observed behavior can also occur for other reasons, such as competitive adsorption, changes in adsorbent properties, or specific interactions between the adsorbate and the adsorbent. The observed phenomenon needs further study.

### 3.2. The Effect of Dosage

**Figure 3:**
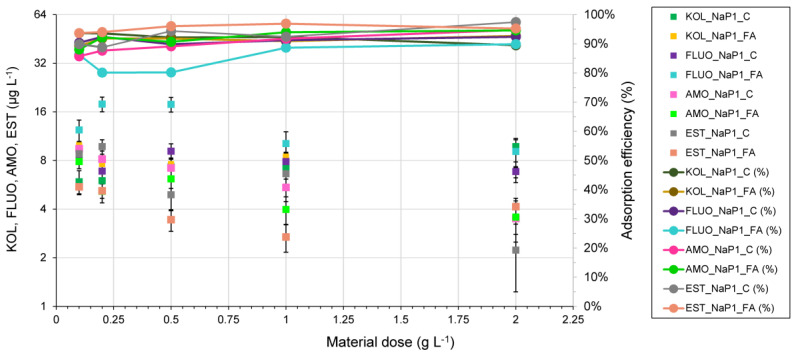
Effect of dose on sorption rate; the initial concentration of each PhCs 100 µg L^−1^.

The use of sorbent-to-solution ratios of 1.0 and 2.0 g L^−1^ translated to the greatest extent in the effective removal of PhCs from solutions (Figure 3). Initial concentrations of FLUO, AMO, and EST of 100 μg L^−1^ reduced to 6.8 μg L^−1^, 3.5 μg L^−1^, and 2.2 μg L^−1^, respectively, after sorption for 2 g NaP1_C L^−1^. Sorbing KOL on NaP1_C, the best removal efficiency was achieved using a dose of 0.1 g L^−1^, while NaP1_FA was most effective using a dose of 2.0 g L^−1^. After applying 0.1 g L^−1^, NaP1_C, KOL concentrations decreased from 100 μg L^−1^ to 5.9 μg L^−1^, and for 2.0 g L^−1^, NaP1_FA to 6.8 μg L^−1^. In contrast, FLUO and KOL concentrations after adsorption on NP1_FA using a dose of 2.0 g L^−1^ decreased to 9.1 μg L^−1^ and 3.6 μg L^−1^, respectively. Applying a dose of 1.0 g NaP1_FA L^−1^ results in the most effective removal of EST, where the concentration after adsorption is 2.7 μg L^−1^. The lowest concentrations obtained after the adsorption reaction are (a) 5.9 μg KOL L^−1^ for a dose of 0. 1 g NaP1_C L^−1^, (b) 6.8 μg FLUO L^−1^ for a dose of 2.0 g NaP1_C L^−1^, (c) 3.5 μg AMO for doses of 2.0 NaP1_C and NaP1_FA g L^−1^, (d) EST—2.7 μg EST for 1.0 g NaP1_FA L^−1^. The results indicate practical sorbent doses that can be used for PhCs removal. They also confirm the results of analyses of the effect of initial concentration on the amount of sorption. The order of PhCs removal efficiency decreases according to the order EST > AMO > FLUO > KOL for comparable sorbent doses.

### 3.3. The Effect of Time Reaction

Pharmaceuticals are effectively removed after 2 min of reaction. Four kinetic models were analyzed to determine the sorption mechanism of selected PhCs on NaP1_C and NaP1_FA sorbents: pseudo-first-order (PFO), pseudo-second-order (PSO), the Behnajady-Modirshahla-Ghanbary (BMG), and Weber’s intraparticle diffusion (WID) model. Details of kinetic models are presented in Supplementary Material (Text S2). The coefficients of determination (R^2^) used to measure the quality of model fit and kinetic parameters are presented in Table 2.

The statistical analyses showed that the sorption kinetics of all analyzed PhCs on NaP1_C and NaP1_FA should be interpreted using the PSO and BMG kinetic model [50]. The magnitudes of the R^2^ parameter for both models above took values close to 1.

**Figure 4:**
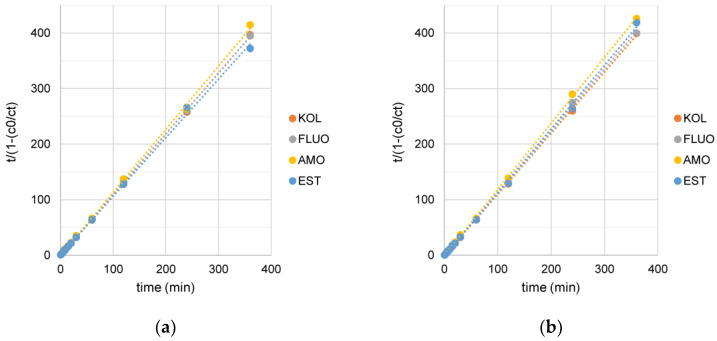
BMG kinetic models for the adsorption of PhCs on (**a**) NaP1_C and (**b**) NaP1_FA; the initial concentration of PhCs: 93 µg KOL L^−1^, 90 µg FLUO L^−1^, 64 µg AMO L^−1^, 85 µg EST L^−1^.

Plotting the curves (Figure 4) of the relationship t/(1 − (c_0_/c_t_)) from t for the BMG kinetic model, where *c_t_* is the concentration of PhCs at time t (µg L^−1^), t is the duration of the experiment (min), *c_0_* is the initial concentration of PhCs (µg L^−1^), allowed to determine the theoretical maximum degradation of each PhCs. According to the assumptions given in [50], the inverse of the parameter *b*, the slope of the straight line to the abscissa axis, was used.

Using NaP1_C sorbent theoretically reduces the initial concentrations of PhCs from 88% (for AMO) to 95% (EST). Realistic AMO and EST sorption efficiency results are very similar and differ from model data by 0% to 3%. For NaP1_FA, the determined theoretical sorption efficiency ranges from 87% (EST) to 91% (KOL). The actual sorption efficiency results range from 91% for AMO to 97% for EST. The most significant differences between the estimated sorption volume and the results obtained in experiments are shown by AMO and EST. The discrepancies are 7% and 10%, respectively. Regardless of the sorbent used and which PhCs were analyzed, the real sorption volume gave better values than the model data. 

As mentioned in the article by [40], the BMG kinetic model is not commonly used. Most commonly, the sorption kinetics of pharmaceuticals are described using the PSO kinetic model, which has been used for adsorbents such as modified zeolites [44,51]. Our results confirm that PSO describes the sorption of PhCs on zeolites very well (Figures S17–S24). The high correlation coefficient R^2^ and the very good projection of the calculated qe value of the accurate sorption results (Table 2) indicate the abundance of active sites on the surface and in the pores of the investigated samples. No less [52] analyzed the kinetics of the oxidative breakdown of EST in water. This process was driven by reactive oxygen species generated from the decomposition of H_2_O_2_, aided by polyvinylpyrrolidone (PVP)-stabilized silver nanoparticles (AgNPs). They employed the BMG kinetic model for their study.

The WID model of KOL and FLUO adsorption suggests that the process involves two stages (Figures S25–S28). During the film diffusion stage, KOL and FLUO molecules initially move from the solution to the outer surface of NaP1_C and NaP1_FA. The molecules undergo intramolecular diffusion, gradually moving to active binding sites within the pores [53]. As Table 2 shows, the slope (k_i_) of the initial stage is significantly steeper than that of the latter stage, highlighting the gradual nature of intramolecular diffusion [54]. Notably, the fitted lines don’t intersect the origin, indicating that multiple processes, not only intramolecular diffusion, influence the adsorption rate [55]. Additionally, the intersection point (c) indicates the boundary layer effect, with a larger c_2_ than c_1_, suggesting that the second adsorption step significantly impacts the rate-limiting stage [55]. According to the WID model, AMO and EST sorption would occur through a single step (Figures S29–S32).

### 3.4. The Effect of pH

**Figure 5:**
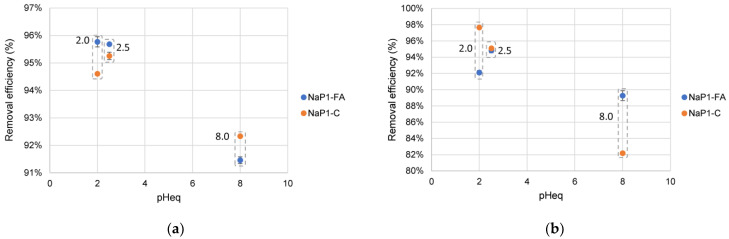
The effect of pH on the removal efficiency of KOL (**a**) and FLUO (**b**). The initial concentration of each is PhCs 100 µg L^−1^, and the adsorbent dosage is 1.0 g L^−1^. Boxes marked with a dotted line indicate results for NaP1_FA and NaP1_C for the same initial pH values.

Figure 5 shows the experimental results of the solutions’ initial pH on KOL and FLUO removal efficiencies. The studies were performed for these two pharmaceuticals because all PhCs have similar removal efficiencies for varying initial concentrations. Also, KOL and FLUO differ in their physicochemical properties (Table 1).

The sorption efficiency of KOL on NaP1_FA and NaP1_C reached values above 90% regardless of pH, with higher values for pH 2–2.5 (95–96%) than pH 8 (92%). There was no significant relationship between the amount of sorption at given pH conditions and the type of sorbent. FLUO was sorbed with an efficiency above 80%. The most effective in reducing FLUO concentration was NaP1_C at pH 2 and 2.5, for which the sorption efficiency was 98 and 95%, respectively. NaP1_FA adsorbed 92% and 95% of the initial FLUO concentration under the same pH conditions. An increase in pH to 8 resulted in a slight decrease in FLUO sorption efficiency to 90% on NaP1_FA and a significant decrease to 82% when NaP1_C was applied. In an acidic environment, KOL becomes more positively charged due to the protonation of its amino groups. This enhances its binding to zeolites, and organic carbon exists in both sorbents. Also, FLUO’s secondary amine is protonated, making the molecule positively charged. This can increase its solubility in water and affect its interaction with sorbents [56]. Zeolite P1, the dominant component of both sorbents, carries a negative charge and may effectively sorb positively charged molecules of FLUO and KOL. Both molecules become less charged in basic conditions. KOL’s amino groups and FLUO’s secondary amine lose protons, reducing their ionic character and decreasing their sorption efficiency.

In the experiment conducted, pH conditions similar to those prevailing in typical municipal wastewater treatment plant leachate (pH = 8.0) were simulated. The sorption efficiencies of KOL and FLUO on both tested sorbents are above 80%, which can be considered satisfactory from the practical use of the sorbents. [40] evaluated the removal efficiency of erythromycin using the same zeolite sorbents as in the present study. For an initial pH = 8.0, better sorption efficiencies were obtained for NaP1_C than for NaP1_FA, which were 81% and 29%, respectively. [57] analyzed the effect of pH on KOL removability in wastewater using Ferrate (VI) Oxidation. The degree of KOL degradation took values ranging from 88.7% (pH 10.0) to 96.6% (pH 7.0). [58]. On the other hand, KOL degradation was reportedly higher in wastewater for pH 7.0 than for 5.0, and after 24 h, it reached up to 80%. FLUO also has the best removability from wastewater at pH 7.0 [59].

Regardless of the sorption mechanism and the influence of the physicochemical properties of the sorbate solutions, zeolites synthesized from fly ash have sorption properties against PhCs comparable to or even better than other mineral and organic sorbents (Table 3). Combined with the economically reasonable cost of synthesizing this type of sorbent, such properties seem to be an incentive for their use in removing PhCs from aqueous solutions.

### 3.5. FTIR Results

**Figure 6:**
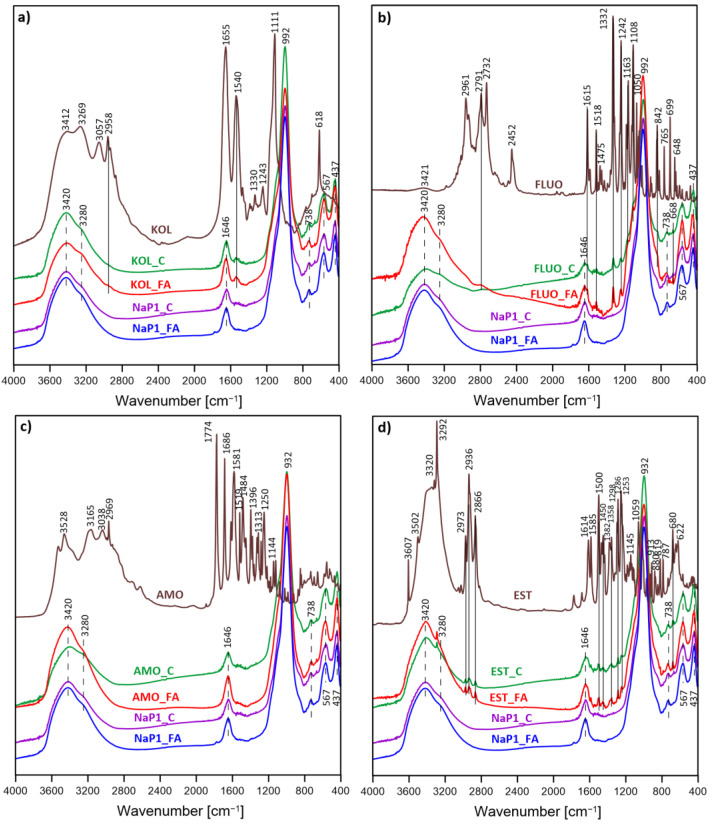
FTIR spectra of NaP1_FA and NaP1_C before and after reaction with (**a**) KOL (KOL_FA, KOL_C), (**b**) FLUO (FLUO_FA, FLUO_C), (**c**) AMO (AMO_FA, AMO_C), (**d**) EST (EST_FA, EST_C) as well as spectra of pure PhCs; the initial concentration of each PhCs 200 mg L^−1^, an adsorbent dosage 1.0 g L^−1^.

Figure 6 presents the spectra of NaP1_FA and NaP1_C both before and after their reaction with PhCs, alongside the spectra of raw PhCs. A notable broad band ranging from 3600 to 3100 cm^−1^ for NaP1_FA and NaP1_C before they react with PhCs is indicative of the stretching vibrations of hydroxyl groups from the water molecules absorbed in the zeolite channels [78,79]. The materials exhibit distinct bands at around 1644 and 738 cm^−1^, linked to the bending vibrations of H–OH and the stretching vibrations of Si–O–Si/Si–O–Al bonds within the tetrahedra. The band at 567 cm^−1^ points to the mullite admixture derived from the fly ash used to synthesize zeolite NaP1. The band at 738 cm^−1^ is also attributed to Si–O–Si/Si–O–Al bending vibrations [80]. The TO_4_ (T = Si or Al) tetrahedral in zeolite P1 shows a band vibration at 437 cm^−1^ [81]. Notably, a sharp, high-intensity band at 992 cm^−1^ confirms the formation of a well-defined aluminosilicate skeleton, attributable to the asymmetric stretching vibrations of the zeolite frameworks’ bridge bonds Si–O(Si) and Si–O(Al). 

The FTIR spectrum of pure KOL (Figure 6a) shows a broad band at ~3412 cm^−1^, linked to the stretching vibrations of –OH and –NH groups. Additionally, it displays characteristic bands for amide I (C=O stretching) and amide II (N–H bending) at 1655 and 1540 cm^−1^, respectively [82]. The peaks at 3269 cm^−1^ and 3057 cm^−1^ are attributed to amide A and B, respectively, due to the N–H stretch in resonance with the amide II overtone [83]. Various C–H stretching vibrations appear around 2900 cm^−1^ [83], with a dominant band at 2958 cm^−1^. A band at 1111 cm^−1^ is due to the antisymmetric stretching of the sulfate counter ion. After KOL sorption, the spectra of NaP1_FA and NaP1_C (Figure 6a) reveal two new bands compared to the raw NaP1_FA and NaP1_C spectra. A relatively strong band at 1540 cm^−1^ and a very weak band at 2958 cm^−1^. These correspond to the amide II (N–H bending) and C–H stretching vibrations. Their presence at the same positions as those in raw KOL indicates the physical sorption of KOL on NaP1_FA and NaP1_C, highlighting the interaction between KOL and the zeolite sorbents.

The spectrum of FLUO (Figure 6b) highlights the complexity of structural information derived from FTIR bands. At 3421 cm^−1^, we observe the N–H group stretching vibration. A band at 2961 cm^−1^ reveals C–H stretching vibrations and C–C ring modes. Bands at 1108 and 1050 cm^−1^ showcase C–H in-plane bending vibrations, while a band at 842 cm^−1^ indicates out-of-plane bending vibrations of the C–H ring. The 1615 cm^−1^ band is linked to C=C stretching vibrations, and the 1518 cm^−1^ band also pertains to C=C stretching. A band at 1475 cm^−1^ indicates C–C–H bending vibrations. The 1332 cm^−1^ and 1242 cm^−1^ bands are characteristic of C–F stretching vibrations from the trifluoromethyl group. Additionally, a band around 699 cm^−1^ is typical of phenyl ring vibrations in mono-substituted FLUO, with ring deformation noted at the 648 cm^−1^ band. The prominent FTIR features align with previously reported FTIR spectroscopic data [84,85,86]. Changes in the FTIR spectrum confirm successful FLUO loading on NaP1_FA and NaP1_C surfaces. Distinct bands at 1332 cm^−1^ and 1242 cm^−1^ (C–F stretching vibrations) and 1518 cm^−1^ (C=C stretching vibrations) signal the presence of FLUO functional groups post-adsorption by zeolite sorbents. Bands associated with C–F bonds underscore the active involvement of trifluoromethyl groups in binding FLUO to NaP1_FA and NaP1_C. Noticeable bands around 842 cm^−1^ (C–H out-of-plane bending vibrations) and 2791 cm^−1^ (aromatic chain presence) further substantiate this. The FLUO spectrum’s 648 cm^−1^ band shifting to 668 cm^−1^ in the FLUO_FA and FLUO_C spectra suggests a robust bond between FLUO and the sorbents. Figure 6c showcases the spectra of pure AMO and zeolite sorbent samples following AMO sorption. The bands observed between 3500 and 3200 cm^−1^ indicate O–H bonds. The aromatic ring contributes to the weak C–H stretching mode bands at 3038 cm^−1^. A band at 2969 cm^−1^ is associated with the stretching vibration of aliphatic C–H groups [87]. Another distinctive band appears at 1686 cm^−1^, corresponding to secondary amides [88]. Bands at 1581, 1519, and 1484 cm^−1^ are linked to the symmetric and asymmetric vibrations of the COO group in the AMO molecule [89]. Intense bands at 1250 cm^−1^ are attributed to the vibrations of the secondary amide’s C–N and N–H bonds [90]. Additionally, the band at 1774 cm^−1^ corresponds to C=O vibrations, and the band at 1144 cm^−1^ corresponds to symmetric S=O stretching in sulfonamide vibrations. No significant spectral changes were observed in NaP1_FA and NaP1_C after AMO sorption.

The FTIR spectrum of EST is depicted in Figure 6d. According to literature data, several distinctive bands are identified for the infrared spectral analysis of EST, particularly when the compound is adsorbed from an aqueous medium [91]. The stretching vibrations of the O–H groups in the EST structure appear as a broad band in the 3650–3100 cm^−1^ range, indicating significant H-bonding in the solid state, overlapping with the sharp band of surface-adsorbed water [92]. Specific bands are observed at 3607, 3502, and 3292 cm^−1^. The strong band at 3320 cm^−1^ is attributed to the (≡C–H) stretch from the alkyne moiety. The symmetric and asymmetric stretching vibrations of other C–H bonds, including those from the CH_3_ and CH_2_ groups, are represented by bands at 2973, 2936, and 2866 cm^−1^. Bands at 1614, 1585, 1500, and 1450 cm^−1^ are due to symmetric C–C stretching (C–C and C=C) and bending (CH_3_ and CH_2_). Bands at 1382, 1358, 1298, and 1286 cm^−1^ are due to symmetric methyl bending (CH_3_) and hydroxyl bending (COH). Bands at 1253 and 1059 cm^−1^ are likely due to C–O stretching vibrations. Other bands, recorded at 1145, 913, 880, 819, 787, 680, and 622 cm^−1^ in the fingerprint region, are due to skeleton vibrations and various combination bands, which require theoretical vibrational spectra simulations using density functional theory for accurate attribution [93]. The spectra of NaP1_FA and NaP1_C samples post-EST sorption revealed additional bands: 2973, 2936, and 2866 cm^−1^ corresponding to C–H bonds in EST, 1500 and 1450 cm^−1^ corresponding to C–C bonds, 1358 and 1286 cm^−1^ assigned to CH_3_ and COH bonds, respectively, and 1253 cm^−1^ corresponding to C–O bonds. The appearance of these bands in the spectra of samples after EST sorption, matching the positions in pure EST, suggests that EST is adsorbed through physical sorption.

### 3.6. Regeneration

**Figure 7:**
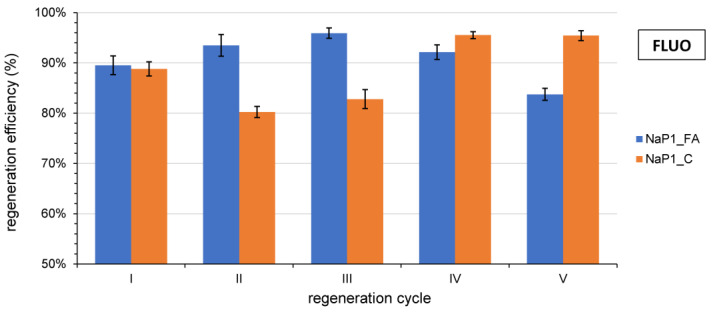
The effectiveness of FLUO sorption in NaP1_FA and NaP1_C after multiple regeneration cycles ([47], changed).

Differences in the strength of methanol-analyte interaction for individual pharmaceuticals depend primarily on the desorbed substance’s chemical properties and the sorbent-analyte bond’s strength. An example is an ethanol desorption experiment that was performed only for FLUO. Figure 7 illustrates the notable differences in the desorption capacity of FLUO when using methanol from the NaP1_FA and NaP1_C sorption materials. Methanol, a polar protic solvent, is widely employed for extraction because of its high polarity. Its volatility allows it to effectively extract and eliminate lipophilic and hydrophilic substances at room temperature. The high efficiency of desorption of FLUO from sorbents occurs practically in the first regeneration cycle. The results show that methanol can be successfully used as a reagent to remove residues of adsorbed pharmaceuticals in sorption materials. 

## 4. Conclusions

Pharmaceuticals frequently enter the environment because wastewater treatment processes don’t fully eliminate them. Drug residues in water can disrupt ecosystems and contribute to the rise of antimicrobial resistance, a major global threat to human and animal health. Thus, effectively removing pharmaceuticals during wastewater treatment is crucial to mitigating their detrimental impacts on the environment, wildlife, and people.

The efficiency of removing pharmaceuticals hinges on several factors, both physical, such as the extent of wastewater treatment, and chemical, relating to the water matrix composition. Yet, the most pivotal aspect of pharmaceutical adsorption lies in choosing the right adsorbent composition and process conditions. Through numerous lab experiments, we have confirmed that the zeolite NaP1_FA and the NaP1_C composites are highly effective in eliminating pharmaceuticals from aqueous solutions.

Both NaP1_FA and NaP1_C adsorbents were shown to be effective in the removal of KOL, AMO, FLUO, and EST. The textural properties of the NaP1_FA and NaP1_C adsorbents make it possible to use these materials for multiple practical applications in water purification processes containing the pharmaceuticals under study. The specific surface area of both sorbents is similar (64.7 m^2^ g^−1^ for NaP1_FA and 61.0 m^2^ g^−1^ for NaP1_C), and their phase composition is dominated by the zeolite NaP1 [40]. Therefore, both sorbents showed similar removal efficiencies for KOL, AMO, FLUO, and EST, regardless of the tested concentration in the 0.1 to 200 mg L^−1^ range. This relationship was also confirmed in studies of the effect of dose and reaction kinetics. Regardless of the applied dose in the 0.1–2.0 g L^−1^ range, the tested sorbents allowed more than 90% removal efficiency of all tested PhCs. NaP1_FA and NAP1_C sorbed PhCs within the first 2 min of the reaction, and the BMG kinetic model can describe the adsorption process well. The pH influences the removal process of pharmaceuticals. The spectral analysis shows that the main process of immobilizing the tested PhCs on zeolite sorbents is predominantly physical sorption. The studies conducted for KOL and FLUO proved that the tested sorbents have about 90% removal efficiency of these pharmaceuticals in an aqueous environment with a pH of 8.0, similar to municipal wastewater treatment plant leachate.

The research allows us to assume that NaP1_FA and NAP1_C adsorbents have great potential for water treatment technologies to remove pharmaceuticals from the aqueous environment as alternative adsorbents.

## Figures and Tables

**Table 1:** Physical-chemical properties of the studied PhCs.

Pharmaceutical/CAS Number ^a^	Molecular Formula	Weight/Exact Mass g mol^−1^	Solubility in Water ^b^ mg L^−1^	Log K_ow_ ^c^	pK_a_ ^d^
KOL/1264-72-8	C_52_H_98_N_16_O_13_	1155.455/ 1154.74992724	283	−2.4	10.23
FLUO/54910-89-3	C_17_H_18_F_3_NO	309.33/ 309.134049	1.7	4.05	9.8
AMO/26787-78-0	C_16_H_19_N_3_O_5_S	365.404/ 365.10454189	958	0.87	7.22
EST/57-63-6	C_20_H_24_O_2_	296.4034/ 296.177630004	6.77	3.67	−1.7

^a^ IUPAC: International Union of Pure and Applied Chemistry; ^b^ Solubility in water at 25 °C (PubChem); ^c^ Log Kow: refers to the octanol-water partition coefficient, also known as log P (PubChem and DrugBank); ^d^ pKa values at 25 °C (DrugBank. Open Data Drug and Drug Target Database, 2014. http://www.drugbank.ca/ (accessed on 23 June 2024).

**Table 2:** Kinetic parameters of PhCs adsorbed on NaP1_C and NaP1_FA.

Model	Parameters	NaP1_C KOL	NaP1_C FLUO	NaP1_C AMO	NaP1_C EST	NaP1_FA KOL	NaP1_FA FLUO	NaP1_FA AMO	NaP1_FA EST
PFO	q_e_ (µg g^−1^) exp.	85.99	82.71	56.91	79.48	86.76	82.26	54.16	56.31
	q_e_ (µg g^−1^) cal.	3.42	7.21	1.98	1.56	3.77	3.09	3.61	2.44
	k_1_ (min^−1^)	0.0034	0.0077	0.0019	−0.0014	−0.0008	0.0032	0.0092	0.0028
	R^2^	0.1534	0.3059	0.0278	0.0075	0.0044	0.0624	0.7318	0.0787
PSO	q_e_ (µg g^−1^) cal.	84.75	82.64	56.50	80.65	84.75	81.30	53.76	53.19
	k_2_ (g (µg min)	−0.0422	0.0542	−0.4476	−0.0177	−0.0232	−0.0344	−0.0532	−0.0113
	R^2^	0.9998	0.9999	0.9993	0.9987	0.9998	0.9999	0.9997	0.998
BMG	1/m	1.9869	2.2952	−22.7273	1.3526	2.2396	−24.0964	−2.3776	−0.7151
	1/b	0.9466	0.8759	0.8811	0.9479	0.9358	0.8748	0.8411	0.8735
	R^2^	0.9999	0.9999	0.9993	0.9987	0.9999	0.9999	0.9997	0.9989
WID	k_i 1_ (µg (g min^1/2^)	−0.7650	8.6716	0.2383	0.1919	23.1320	10.764	0.136	−0.0877
	k_i 2_ (µg (g min^1/2^)	−0.0314	−0.0475	-	-	−0.0294	−0.0918	-	-
	c_1_ (µg g^−1^)	77.21	51.80	53.41	76.95	53.63	57.26	52.78	56.56
	c_2_ (µg g^−1^)	86.04	83.12	-	-	85.85	83.34	-	-
	R_1_^2^	0.7632	0.5634	0.3271	0.1812	0.9810	0.8614	0.0539	0.0224
	R_2_^2^	0.0215	0.0814	-	-	0.0122	0.2590	-	-

**Table 3:** Uptake of KOL, FLUO, AMO, EST by zeolites and other sorbents.

Pharmaceutical	Adsorbent Type	Adsorption Capacity [mg g^−1^]	References
KOL	Sandy loam	4.467	[60]
	Sand	4.467	
	Loam	4.600	
	NaP1_FA	199.3	This work
	NaP1_C	199.3	
FLUO	Synthetic zeolites (Zaolite 13x)	32.11	[61]
	Synthetic zeolites (Zeolite 4A)	21.8	
	Granular activated carbon (GAC)	233.5	
	Spent coffee grounds	14.31	
	Pine bark	6.53	
	Cork waste	4.74	
	NaX/TiO_2_	87.50	[62]
	Hydrochar, activated carbons	44.1	[63]
	Rice bran biochar	67.58	[64]
	Eucalyptus biochar	6.41	[65]
	NaP1_FA	198.3	This work
	NaP1_C	198.2	
AMO	Nanoporous carbon from MCM-22 zeolite	116.1	[66]
	Natural beige sepiolite	100	[67]
	Activated carbon from olive biomass prepared by muffle furnace (ACF)	237.02	[68]
	Activated carbon from olive biomass prepared by microwave-induced (ACMW)	166.96	
	Clay decorated carbon nanocomposite	68.3	[69]
	Almond shell ashes	2.5	[70]
	Chitosan beads	8.71	[28]
	Crosslinked β-cyclodextrin-carboxymethylcellulose polymer	5.076	[71]
	Paper mill sludge-based activated carbon with KOH	191.6	[72]
	Bentonite	53.9315	[73]
	NaP1_FA	206.4	This work
	NaP1_C	206.4	
EST	Nanoporous carbon from MCM-22 zeolite	116.1	[66]
	Surface-modified zeolitic tuff with hexadecyltrimethylammonium 25 mM (HDTMA)	0.7073	[74]
	Nanocomposite of graphene oxide, magnetic chitosan, and organophilic clay (GO/mCS/OC)	50.5	[75]
	Single-walled carbon nanotubes (SWCNTs)	120	[76]
	Na-bent, untreated bentonite	4.07	[77]
	Modification bentonite, NaCl treatment	4.48	
	Fe-Na-bent, Na-bent modified with FeCl_2_∙4H_2_O	5.83	
	NaP1_FA	188.0	This work
	NaP1_C	188.1	

## Data Availability

The original contributions presented in the study are included in the article/Supplementary Materials, further inquiries can be directed to the corresponding author.

## Supplementary Materials

The following supporting information can be downloaded at: https://www.mdpi.com/article/10.3390/ma17153848/s1, Figure S1: Langmuir isotherm plot for the adsorption of KOL on NaP1_FA and NaP1_C; Figure S2: Freundlich isotherm plot for the adsorption of KOL on NaP1_FA and NaP1_C; Figure S3: Dubinin-Radushkevich isotherm plot for the adsorption of KOL on NaP1_FA and NaP1_C; Figure S4: Temkin isotherm plot for the adsorption of KOL on NaP1_FA; Figure S5: Langmuir isotherm plot for the adsorption of FLUO on NaP1_FA and NaP1_C; Figure S6: Freundlich isotherm plot for the adsorption of FLUO on NaP1_FA and NaP1_C; Figure S7: Dubinin-Radushkevich isotherm plot for the adsorption of FLUO on NaP1_FA and NaP1_C; Figure S8: Temkin isotherm plot for the adsorption of FLUO on NaP1_FA; Figure S9: Langmuir isotherm plot for the adsorption of AMO on NaP1_FA and NaP1_C; Figure S10: Freundlich isotherm plot for the adsorption of AMO on NaP1_FA and NaP1_C; Figure S11: Dubinin-Radushkevich isotherm plot for the adsorption of AMO on NaP1_FA and NaP1_C; Figure S12. Temkin isotherm plot for the adsorption of AMO on NaP1_FA; Figure S13: Langmuir isotherm plot for the adsorption of EST on NaP1_FA and NaP1_C; Figure S14: Freundlich isotherm plot for the adsorption of EST on NaP1_FA and NaP1_C; Figure S15: Dubinin-Radushkevich isotherm plot for the adsorption of EST on NaP1_FA and NaP1_C; Figure S16: Temkin isotherm plot for the adsorption of EST on NaP1_FA; Figure S17: The PSO kinetics model for the adsorption of KOL onto NaP1_C; Figure S18: The PSO kinetic model for the adsorption of KOL onto NaP1_FA; Figure S19: The PSO kinetic model for the adsorption of FLUO onto NaP1_C; Figure S20: The PSO kinetic model for the adsorption of FLUO onto NaP1_FA; Figure S21: The PSO kinetic model for the adsorption of AMO onto NaP1_C; Figure S22: The PSO kinetic model for the adsorption of AMO onto NaP1_FA; Figure S23: The PSO kinetic model for the adsorption of EST onto NaP1_C; Figure S24: The PSO kinetic model for the adsorption of EST onto NaP1_FA; Figure S25: The WID kinetic model for the adsorption of KOL onto NaP1_C; Figure S26: The WID kinetic model for the adsorption of KOL onto NaP1_FA; Figure S27: The WID kinetic model for the adsorption of FLUO onto NaP1_C; Figure S28: The WID kinetic model for the adsorption of FLUO onto NaP1_FA; Figure S29: The WID kinetic model for the adsorption of AMO onto NaP1_C; Figure S30: The WID kinetic model for the adsorption of AMO onto NaP1_FA; Figure S31: The WID kinetic model for the adsorption of EST onto NaP1_C; Figure S32: The WID kinetic model for the adsorption of EST onto NaP1_FA; Table S1: Isotherm constants and regression data for used adsorption isotherms. References [94,95,96,97,98,99] are cited in the supplementary materials.
