# Supplementary material for: Using Zeolite Materials to Remove Pharmaceuticals from Water
Source: Materials (Basel). 2024 Aug 3;17(15):3848. doi: 10.3390/ma17153848 (PMC11313275; doi:10.3390/ma17153848)
Supplement: Supplementary file 1 [file materials-17-03848-s001.zip › materials-3100872-supplementary.pdf]

Supplementary Material

Using Zeolite Materials to Remove Pharmaceuticals from Water

Tomasz Bajda [1,*], Agnieszka Grela [2], Justyna Pamuła [2], Joanna Kuc [3], Agnieszka Klimek [1], Jakub Matusik [1], Wojciech Franus [4], Santhana Krishna Kumar A. [5], Tomasz Danek [1] and Paweł Gara [6]

[1] Faculty of Geology, Geophysics and Environmental Protection, AGH University of Krakow, al. A. Mickiewicza 30, 30-059 Krakow, Poland; bajda@agh.edu.pl, aklimek@agh.edu.pl, jmatusik@agh.edu.pl, tdanek@agh.edu.pl
[2] Faculty of Environmental and Power Engineering, Cracow University of Technology, ul. Warszawska 24, 31-155 Krakow, Poland,; agnieszka.grela@pk.edu.pl, justyna.pamula@pk.edu.pl, joanna.kuc@pk.edu.pl
[3] Faculty of Chemical Engineering and Technology, Cracow University of Technology, ul. Warszawska 24, 31-155 Krakow, Poland,; agnieszka.grela@pk.edu.pl, justyna.pamula@pk.edu.pl, joanna.kuc@pk.edu.pl
[4] Faculty of Civil Engineering and Architecture, Lublin University of Technology, ul. Nadbystrzycka 40, 20-618 Lublin, Poland; w.franus@pollub.pl
[5] Department of Chemistry, National Sun Yat-sen University, 70 Lienhai Road, Kaohsiung 80424, Taiwan, R.O.C; krishnakumar@mail.nsysu.edu.tw
[6] Faculty of Mechanical Engineering and Robotics, AGH University of Krakow, al. A. Mickiewicza 30, 30-059 Krakow, Poland; pgara@agh.edu.pl
* Correspondence: bajda@agh.edu.pl

Text 1. Adsorption isotherms

The Langmuir model assumes that all adsorption binding sites are uniform. Additionally, it posits that adsorption forms a single layer and takes place solely on the surface. The model can be expressed in the following linear form [1]:

$$\frac{C_e}{q_e} = \frac{1}{q_{max}K_L} + \frac{C_e}{q_{max}} \qquad (S1)$$

Where Ce (mg L^{-1}) is the equilibrium concentration of PhCs solution; K_L (L mg^{-1}) is the adsorption constant related to the adsorption energy; q_{max} and q_e (mg g^{-1}) are the maximum and equilibrium adsorption capacities, respectively.

The Freundlich isotherm posits that adsorption occurs in a single layer and is reversible. Additionally, it applies to multilayer adsorption on heterogeneous surfaces. This relationship is mathematically represented by [2]:

$$log q_e = log K_F + \frac{1}{n} \cdot ln C_e \qquad (S2)$$

Where n and K_F (mg g^{-1}) represent adsorption intensity and Freundlich constant regarding the adsorption capacity, respectively. In general, the n value indicates the favorability of adsorption. The $n > 1$ represents favorable adsorption.

The Dubinin-Radushkevich (D-R) isotherm model posits that the adsorbent surface is not uniform but varied. Typically, this model is instrumental in assessing the free energy associated with adsorption processes. The corresponding equations are as follows [3]:

$$ln\, q_e = ln q_d - K\varepsilon^2 \qquad (S3)$$

Where q_e (mg g^{-1}) and q_d (mg g^{-1}) represent the amount of adsorbate adsorbed per unit mass of adsorbent at equilibrium and the theoretical monolayer saturation capacity, respectively. K (mol^2 kJ^{-2}) is a constant related to the mean free energy of adsorption per mole of the adsorbate. ε is Polanyi potential, calclutaed as:

$$\varepsilon = RT\, ln(1+\frac{1}{C_e}) \qquad (S4)$$

The Temkin isotherm model posits that as the surface coverage increases, the heat of adsorption for each molecule diminishes in a linear fashion. The equation governing this phenomenon is described by [3]:

$$q_e = B lnA + B lnC_e \qquad (S5)$$

Where q_e (mg g^{-1}) is the amount of adsorbate adsorbed per unit mass of adsorbent at equilibrium, Ce (mg L^{-1}) is the equilibrium concentration of the adsorbate in the solution, A (L g^{-1}) is the Temkin isotherm constant, B (J mol^{-1}) is related to the heat of adsorption (J mol^{-1}).

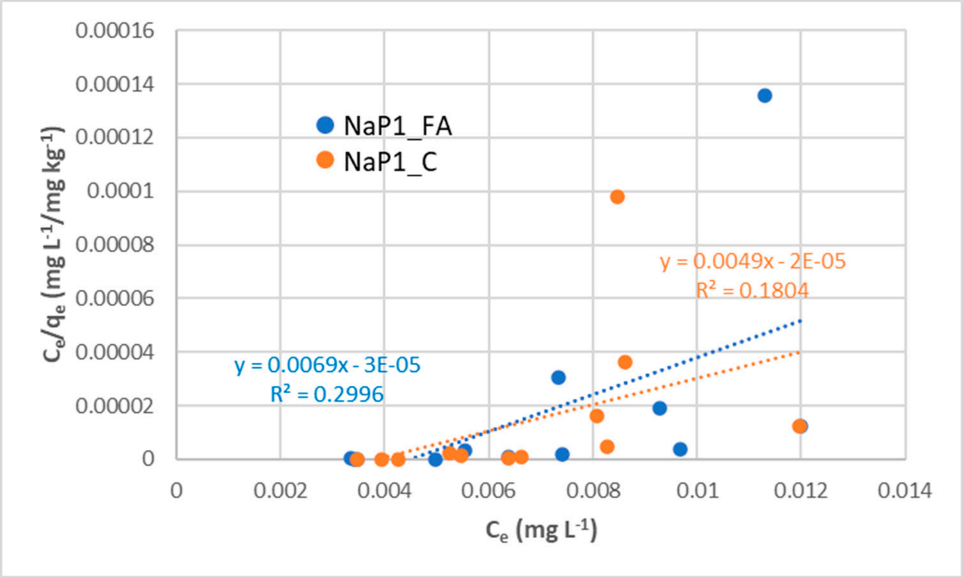

Figure S1. Langmuir isotherm plot for the adsorption of KOL on NaP1_FA and NaP1_C.

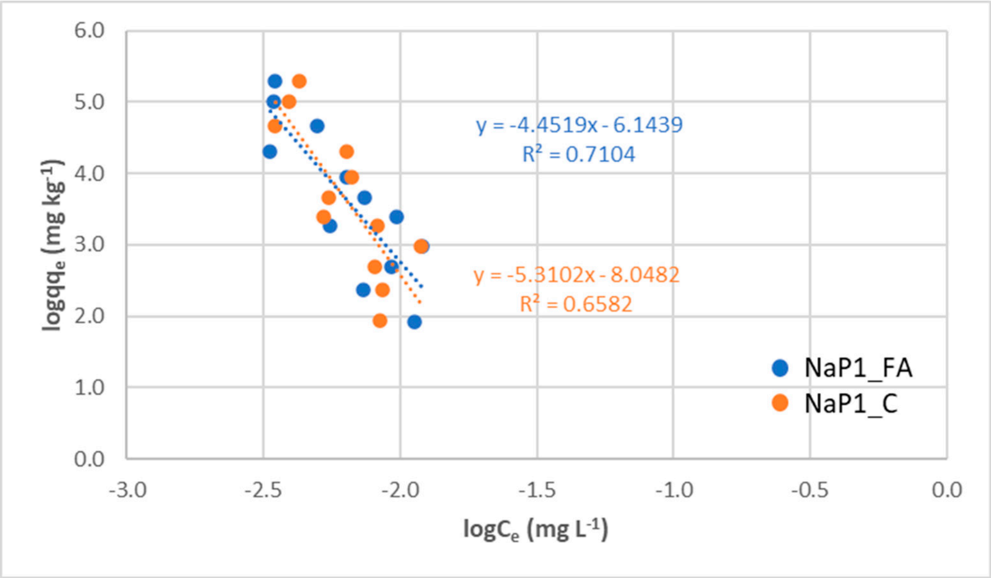

Figure S2. Freundlich isotherm plot for the adsorption of KOL on NaP1_FA and NaP1_C.

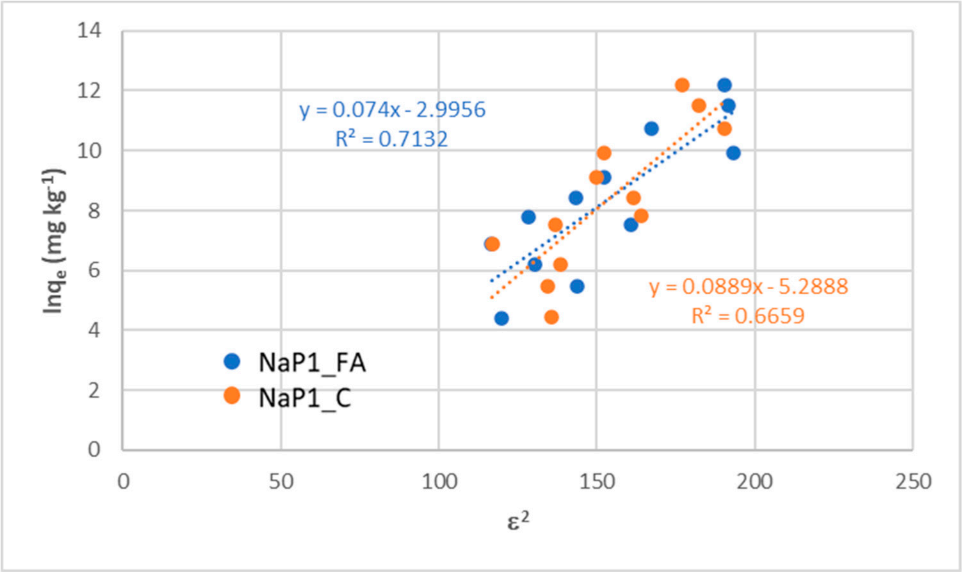

Figure S3. Dubinin-Radushkevich isotherm plot for the adsorption of KOL on NaP1_FA and NaP1_C.

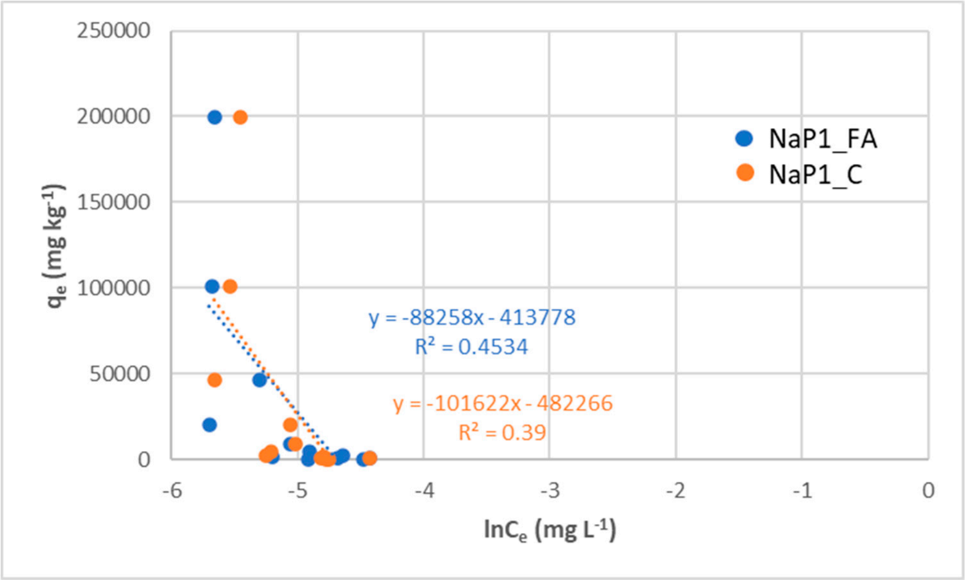

Figure S4. Temkin isotherm plot for the adsorption of KOL on NaP1_FA.

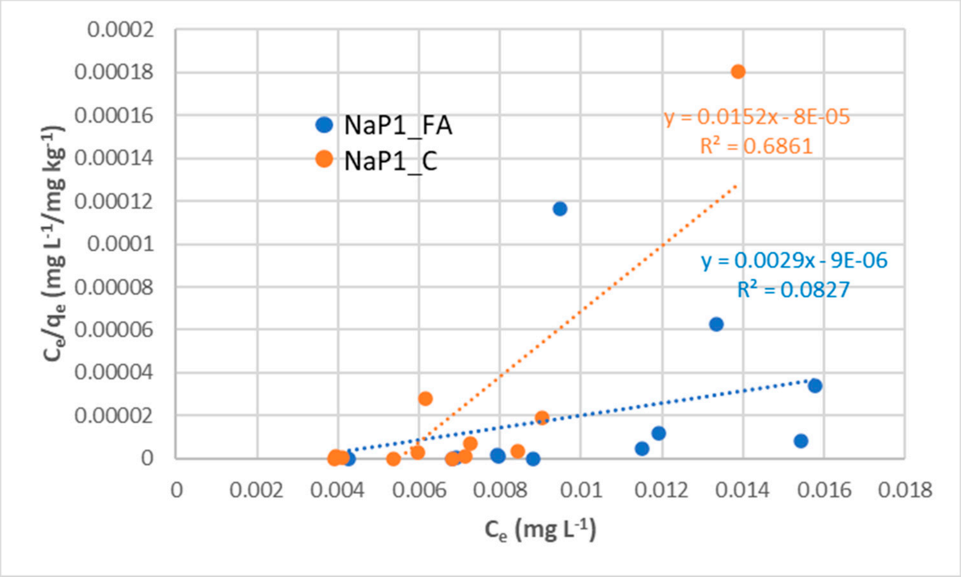

Figure S5. Langmuir isotherm plot for the adsorption of FLUO on NaP1_FA and NaP1_C.

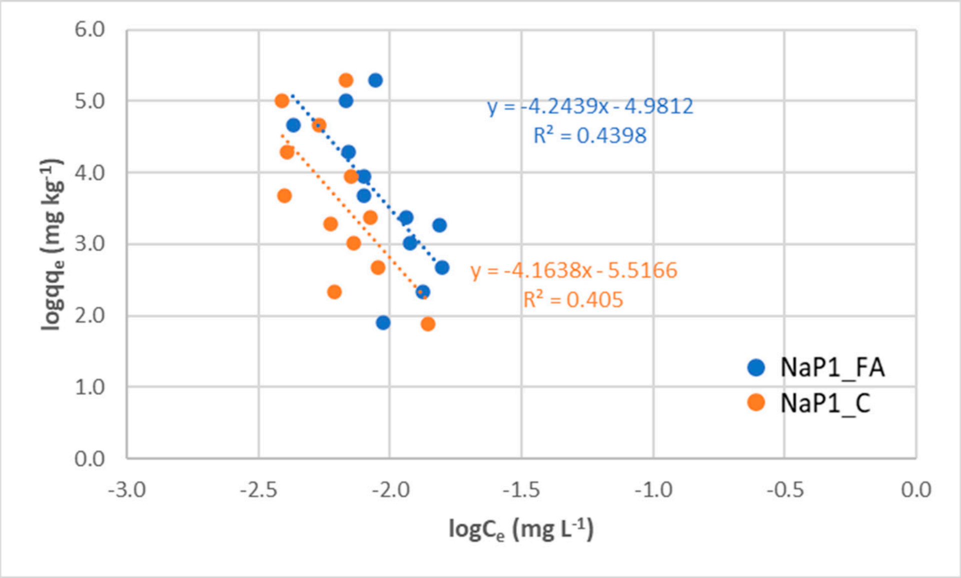

Figure S6. Freundlich isotherm plot for the adsorption of FLUO on NaP1_FA and NaP1_C.

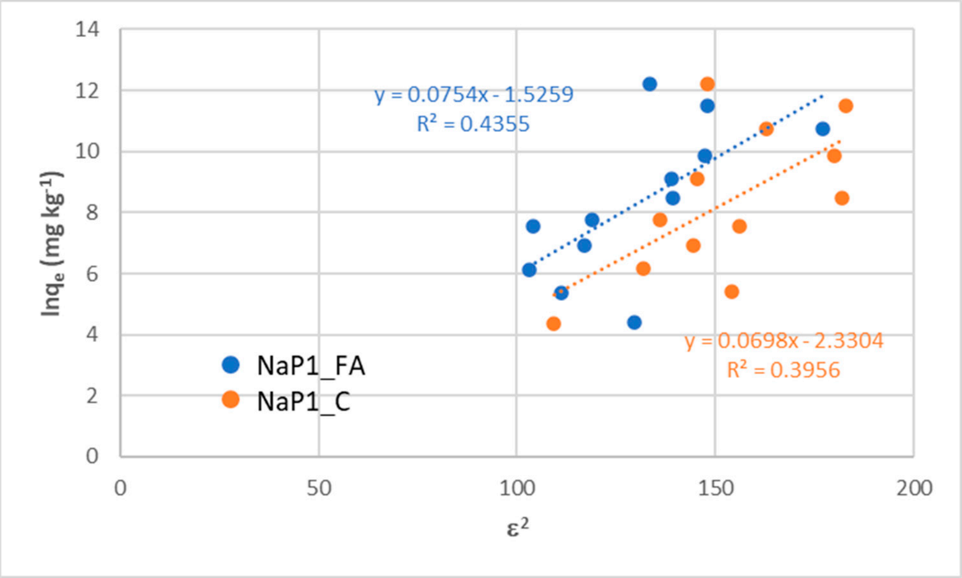

Figure S7. Dubinin-Radushkevich isotherm plot for the adsorption of FLUO on NaP1_FA and NaP1_C.

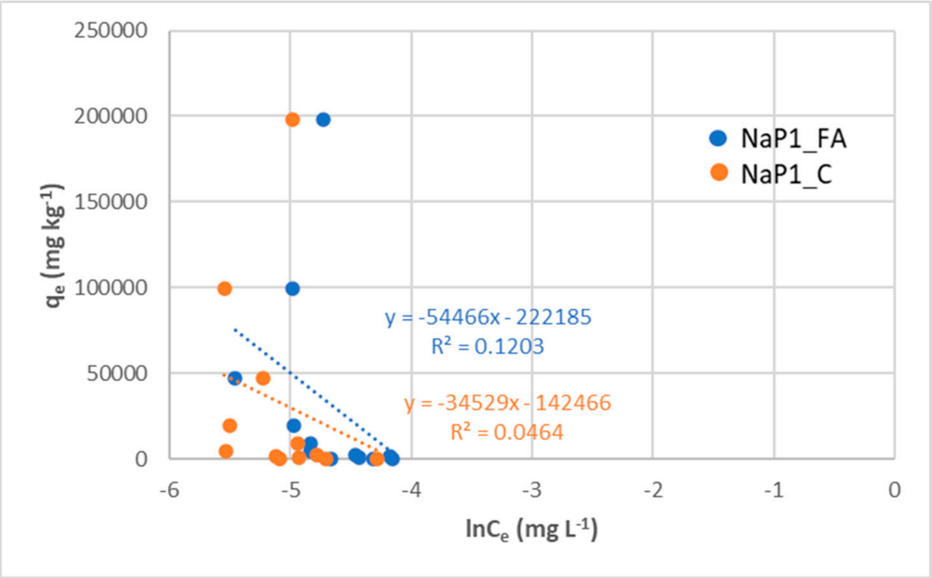

Figure S8. Temkin isotherm plot for the adsorption of FLUO on NaP1_FA.

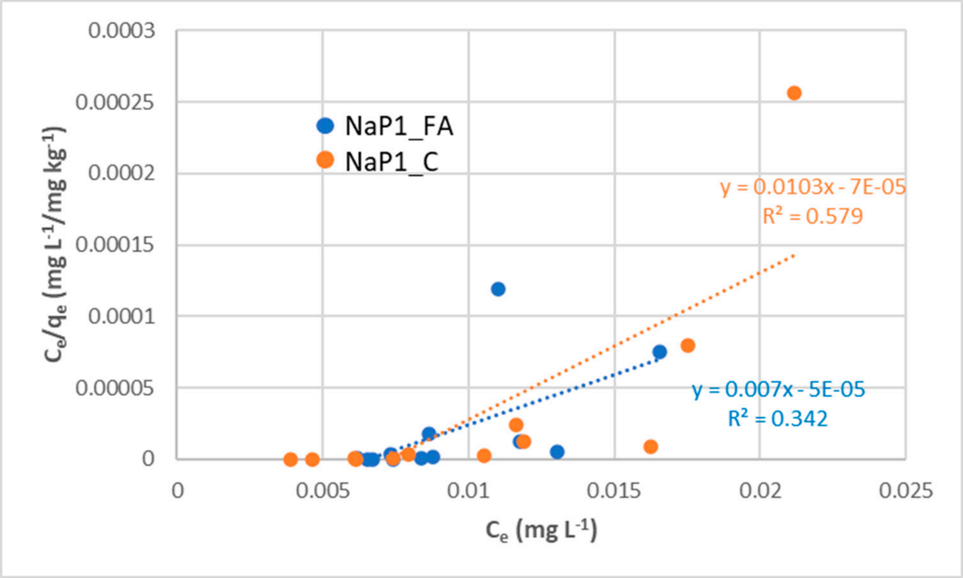

Figure S9. Langmuir isotherm plot for the adsorption of AMO on NaP1_FA and NaP1_C.

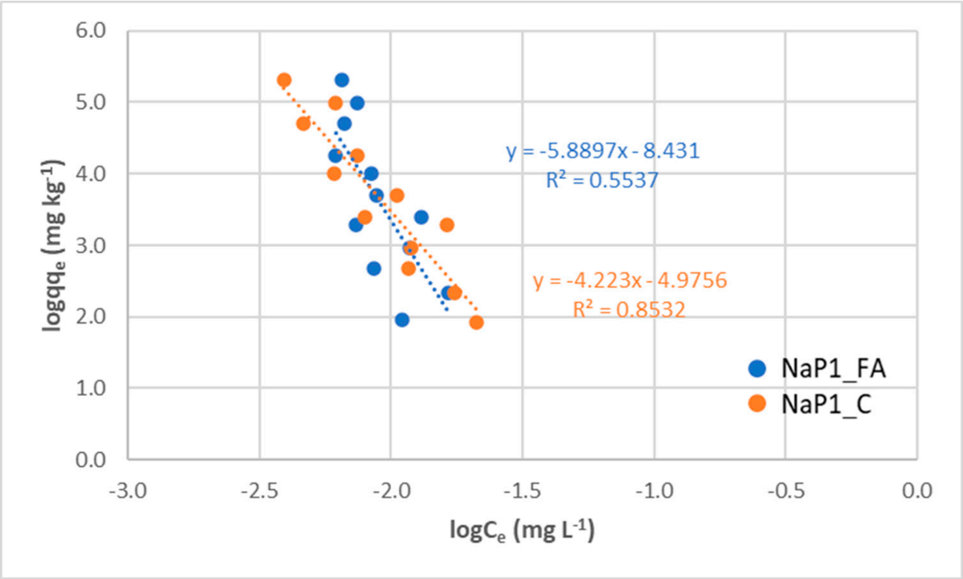

Figure S10. Freundlich isotherm plot for the adsorption of AMO on NaP1_FA and NaP1_C.

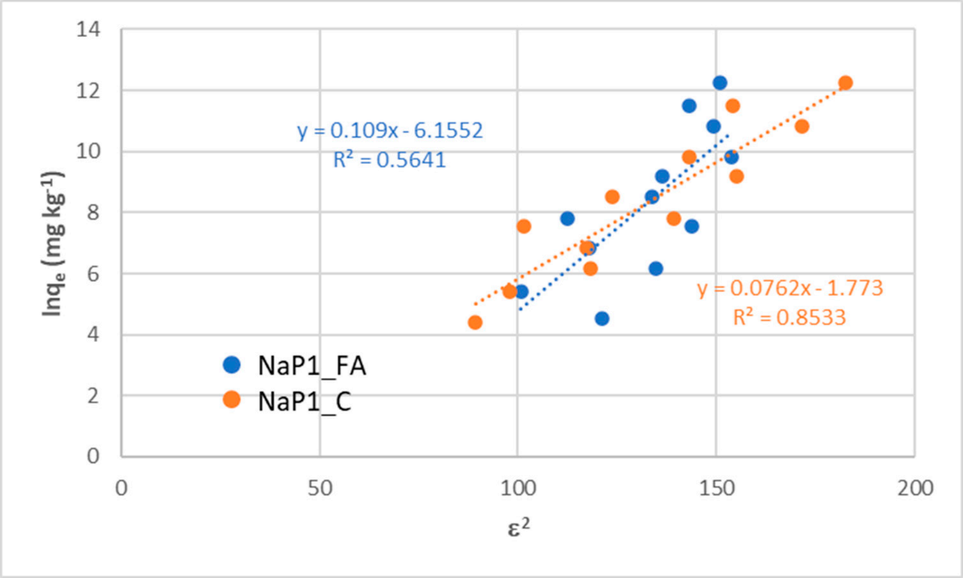

Figure S11. Dubinin-Radushkevich isotherm plot for the adsorption of AMO on NaP1_FA and NaP1_C.

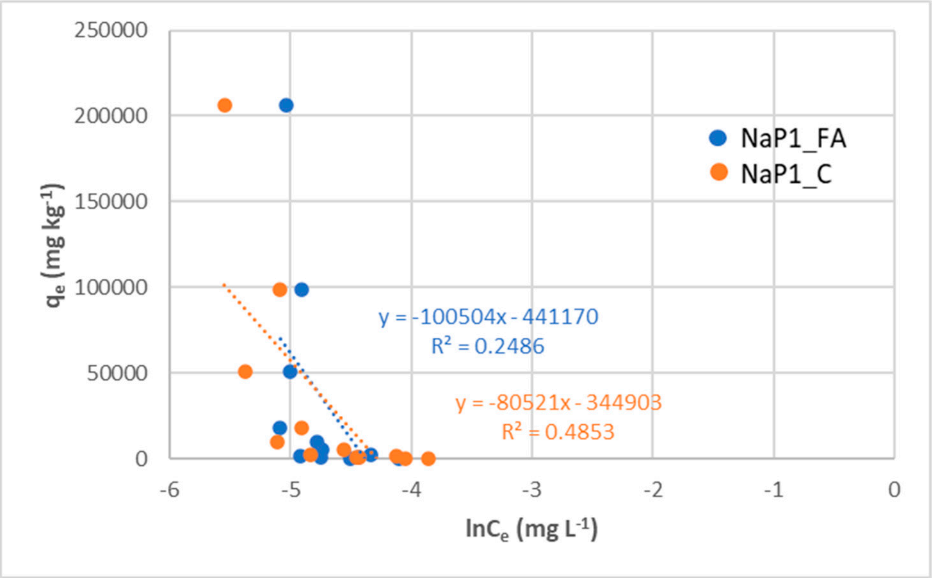

Figure S12. Temkin isotherm plot for the adsorption of AMO on NaP1_FA.

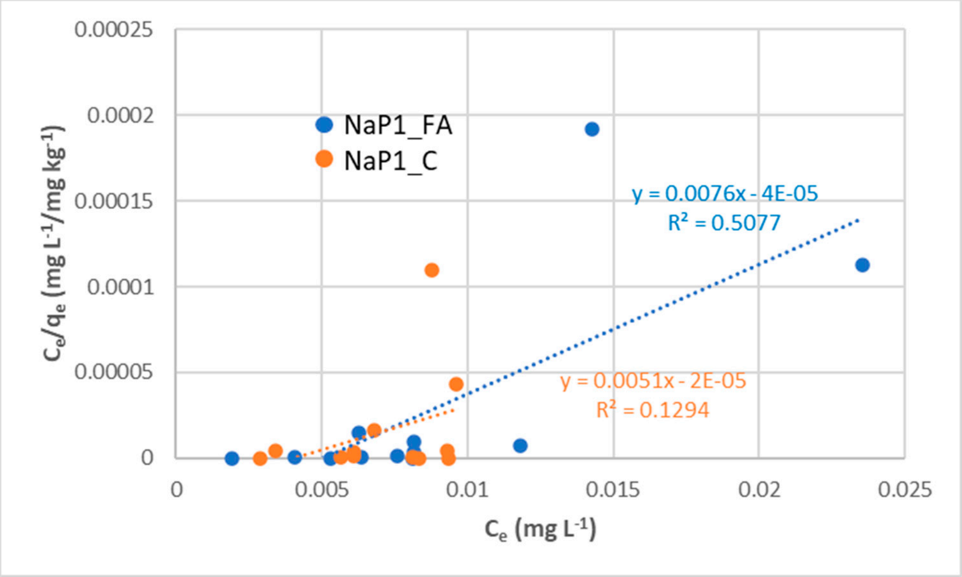

Figure S13. Langmuir isotherm plot for the adsorption of EST on NaP1_FA and NaP1_C.

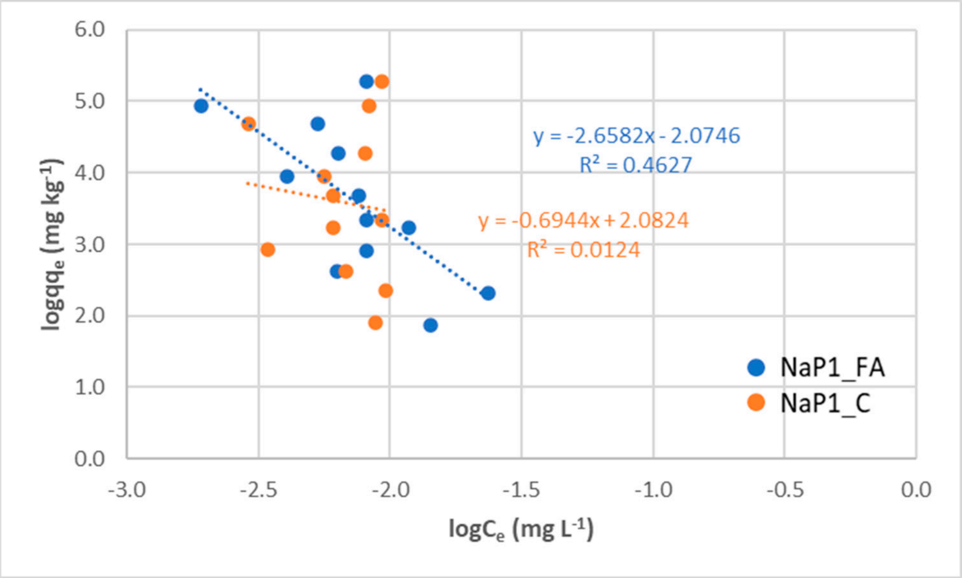

Figure S14. Freundlich isotherm plot for the adsorption of EST on NaP1_FA and NaP1_C.

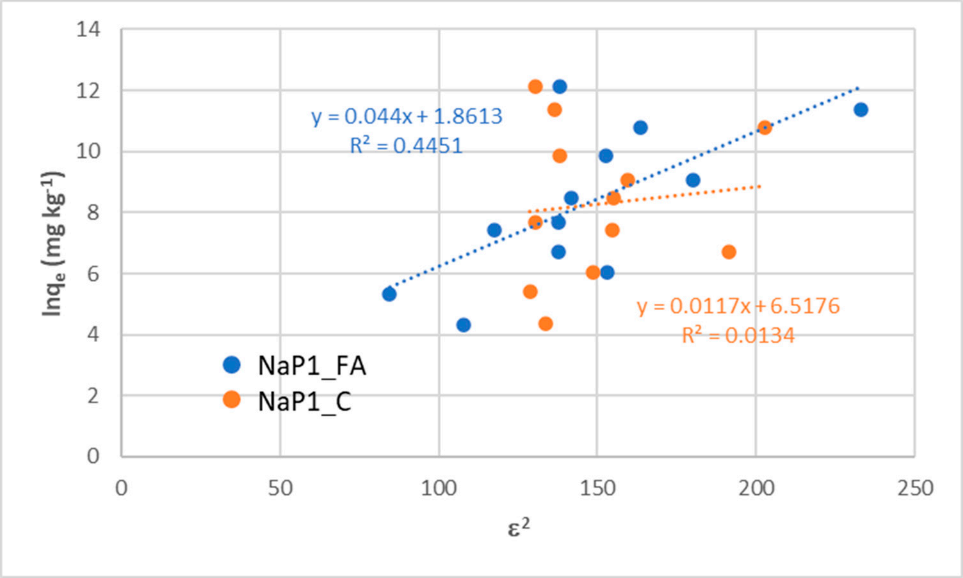

Figure S15. Dubinin-Radushkevich isotherm plot for the adsorption of EST on NaP1_FA and NaP1_C.

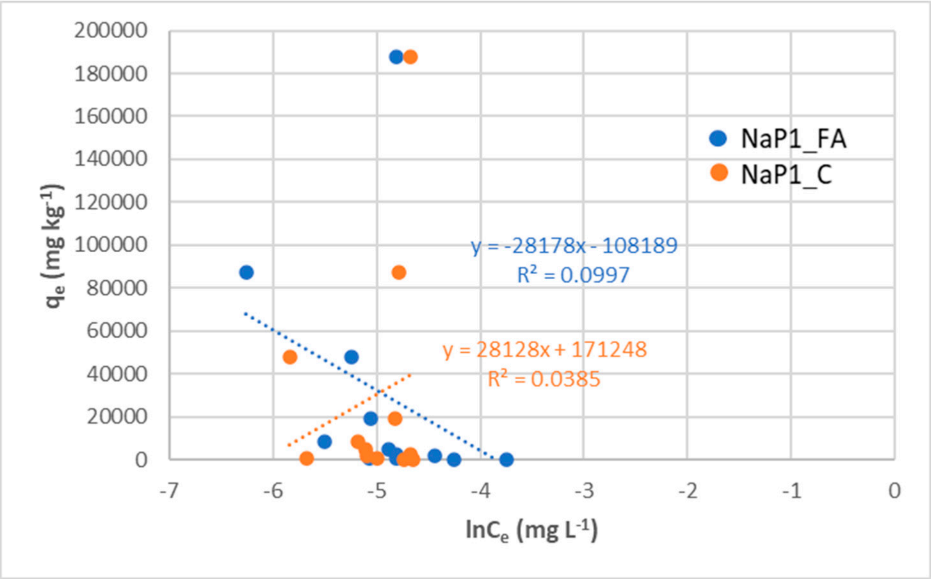

Figure S16. Temkin isotherm plot for the adsorption of EST on NaP1_FA.

Table S1. Isotherm constants and regression data for used adsorption isotherms.

Model	Parameters	NaP1_C KOL	NaP1_C FLUO	NaP1_C AMO	NaP1_C EST	NaP1_FA KOL	NaP1_FA FLUO	NaP1_FA AMO	NaP1_FA EST
Langmuir	q_{max} (mg g^{-1})	204.1	65.8	97.1	196.1	144.9	344.8	142.8	131.6
	K_L (L mg^{-1})	-230	-190	-206	-255	-245	-322	-1000	-190
	R^2	0.2996	0.6861	0.579	0.1294	0.1804	0.0827	0.342	0.5077
Freundlich	n	-0.1883	-0.2402	-0.2368	-1.4401	-0.2246	-0.2356	-0.1698	-0.3762
	K_F (mg kg^{-1})	$8.94\ 10^{-9}$	$3.04\ 10^{-6}$	$1.06\ 10^{-5}$	120.9	$7.18\ 10^{-7}$	$1.04\ 10^{-5}$	$3.71\ 10^{-9}$	0.008
	R^2	0.6582	0.405	0.8532	0.0124	0.7104	0.4398	0.5537	0.4627
R-D	q_d (mg g^{-1})	0.0050	0.0972	0.1698	676.9	0.0500	0.2174	0.0021	6.43
	K (mol^2 kJ^{-2})	-0.0889	-0.0698	-0.0762	-0.0117	-0.074	-0.0754	-0.109	-0.044
	R^2	0.6659	0.3956	0.8533	0.0134	0.7132	0.4335	0.5641	0.4451
Temkin	A (L g^{-1})	115.1	61.9	72.5	440.6	108.7	59.1	80.6	46.5
	B (J mol^{-1})	-101622	-34529	-80521	28128	-88258	-54466	-100504	-28178
	R^2	0.3900	0.0464	0.4853	0.0385	0.4534	0.1203	0.2486	0.0997

Text 1. Adsorption kinetics.

The pseudo-first-order (PFO) kinetics model assumes that the diffusion step primarily controls adsorption, with only a single type of binding site present on the adsorbent surface. The associated formulas are provided below [4]:

$$ln(q_e\text{-}q_t) = lnq_e - k_1t \qquad \text{(S6)}$$

Where q_t and q_e (μg g^{-1}) is the amount of PhCs adsorbed at any time t (min) and equilibrium time, respectively; k_1 (min^{-1}) represents the PFO rate constant.

The pseudo-second-order (PSO) kinetics model is instrumental in determining the adsorption capacity of a sample. This model assumes the existence of two binding sites on the surface of the adsorbent. The linearized form of this model is expressed as follows [5]:

$$t/q_t = 1/k_2q^2_e + t/q_e \qquad \text{(S7)}$$

Where q_t and q_e ($\mu g \cdot g^{-1}$) represent the same meaning as those of Eq. (S1); k_1 ($g \cdot \mu g^{-1} \cdot min^{-1}$) represents the PSO rate constant, which can be utilized to calculate the initial adsorption rate via the limit theorem.

Behnajady-Modirshahla-Ghanbary (BMG) kinetic model could be expressed as follows:

$$t/1\text{-}(C_t/C_o) = m + bt \qquad \text{(S8)}$$

Where Co and Ct are initial and at any time concentration, respectively, and b and m are constants relating to the reaction kinetics.

The adsorption process occurs through the diffusion effects of the adsorbent, which include film, surface, pore diffusions, or any combination of these steps. Weber's intraparticle diffusion (WID) model delved deeper into adsorption. The formula is explained as follows [6]:

$$qt = k_it^{1/2} + c \qquad \text{(S9)}$$

Where k_i ($\mu g \cdot g^{-1} \cdot min^{-1/2}$) is the rate constant of intraparticle diffusion; c (μg/g) is a constant related to the adsorption step.

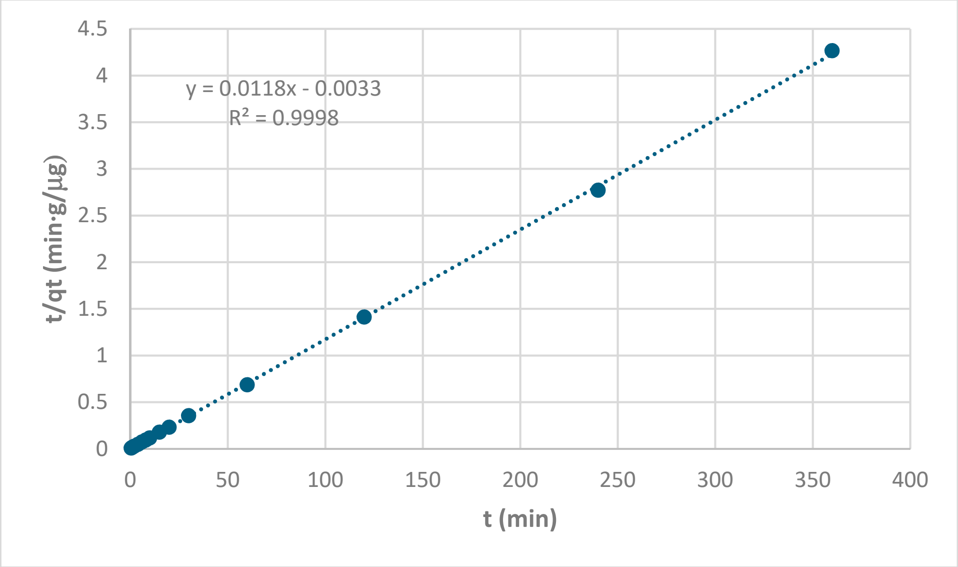

Figure S17. The PSO kinetics model for the adsorption of KOL onto NaP1_C.

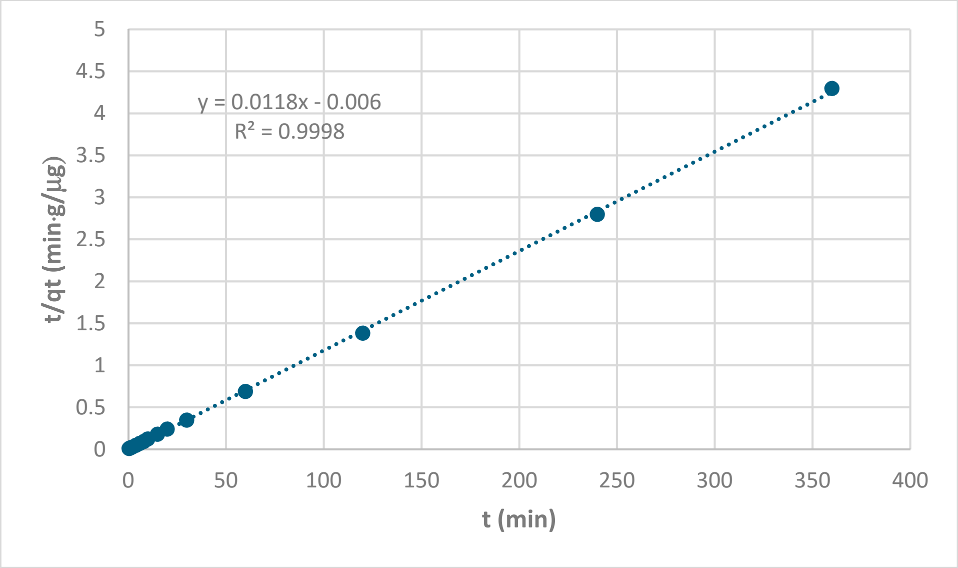

Figure S18. The PSO kinetic model for the adsorption of KOL onto NaP1_FA.

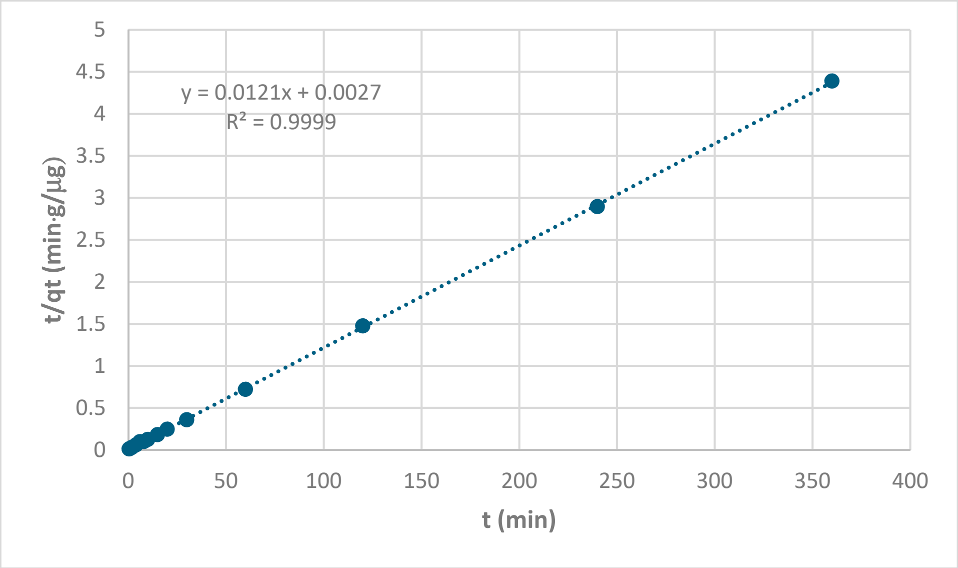

Figure S19. The PSO kinetic model for the adsorption of FLUO onto NaP1_C.

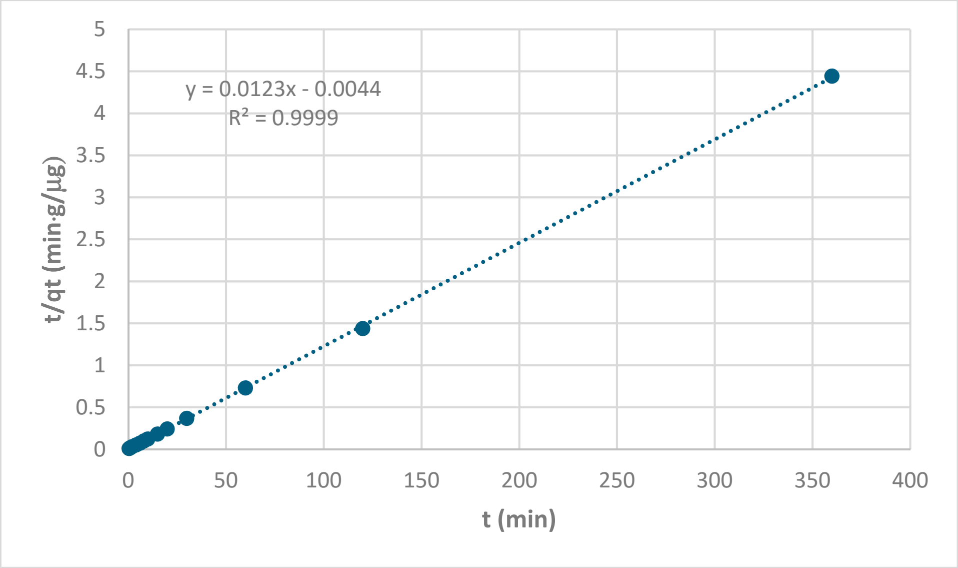

Figure S20. The PSO kinetic model for the adsorption of FLUO onto NaP1_FA.

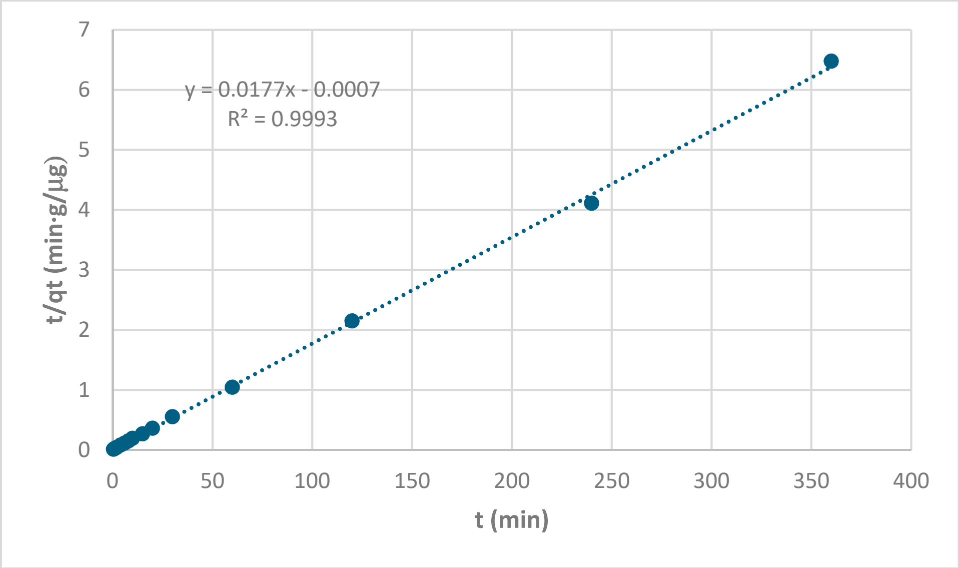

Figure S21. The PSO kinetic model for the adsorption of AMO onto NaP1_C.

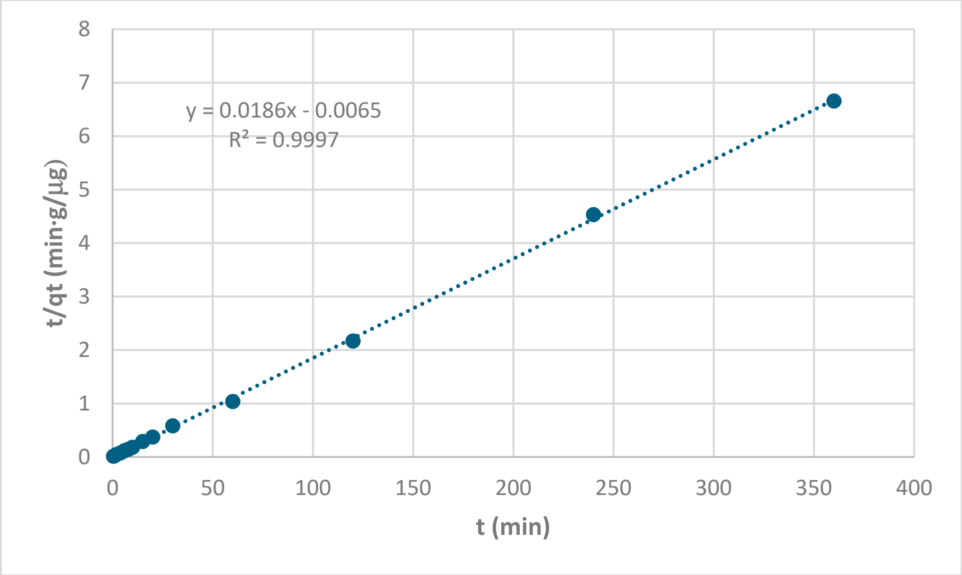

Figure S22. The PSO kinetic model for the adsorption of AMO onto NaP1_FA.

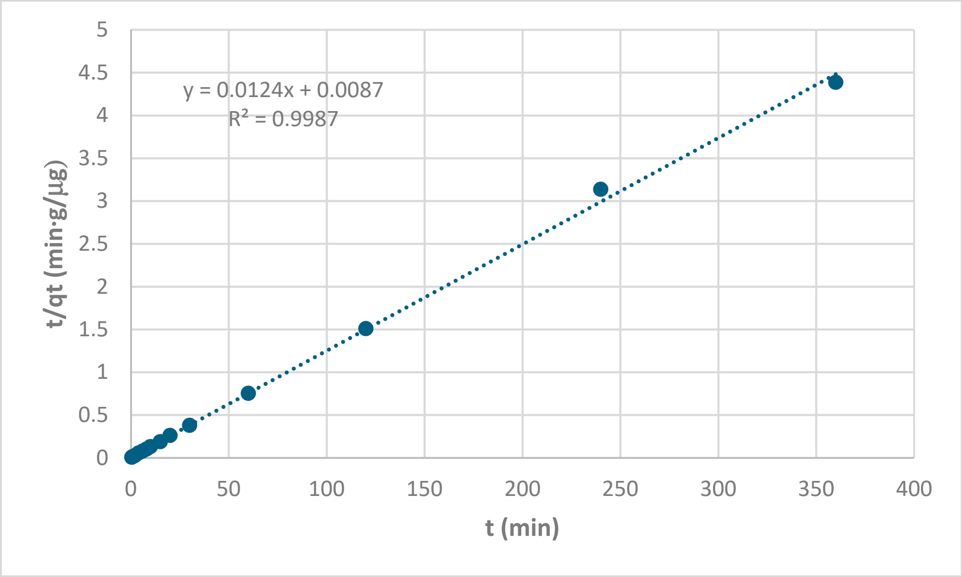

Figure S23. The PSO kinetic model for the adsorption of EST onto NaP1_C.

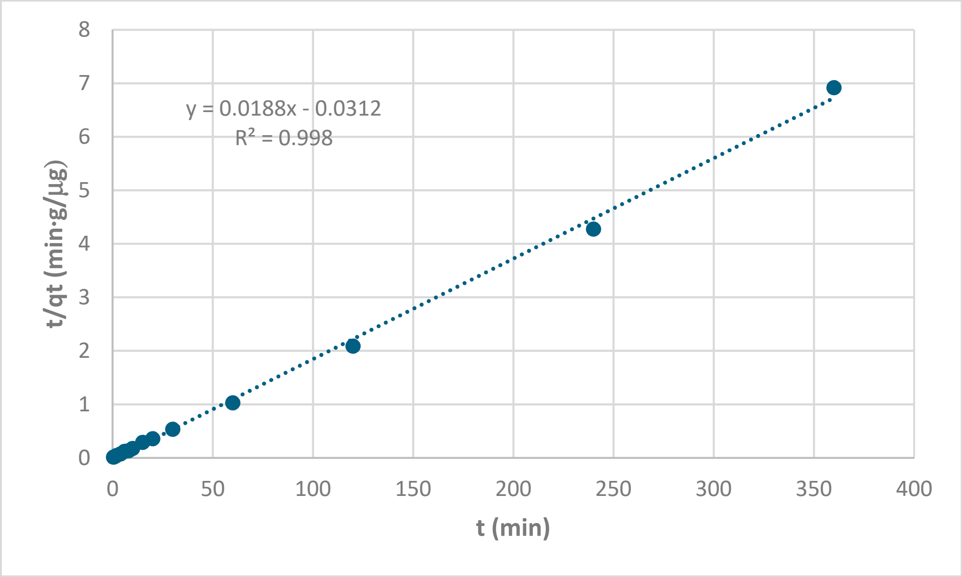

Figure S24. The PSO kinetic model for the adsorption of EST onto NaP1_FA.

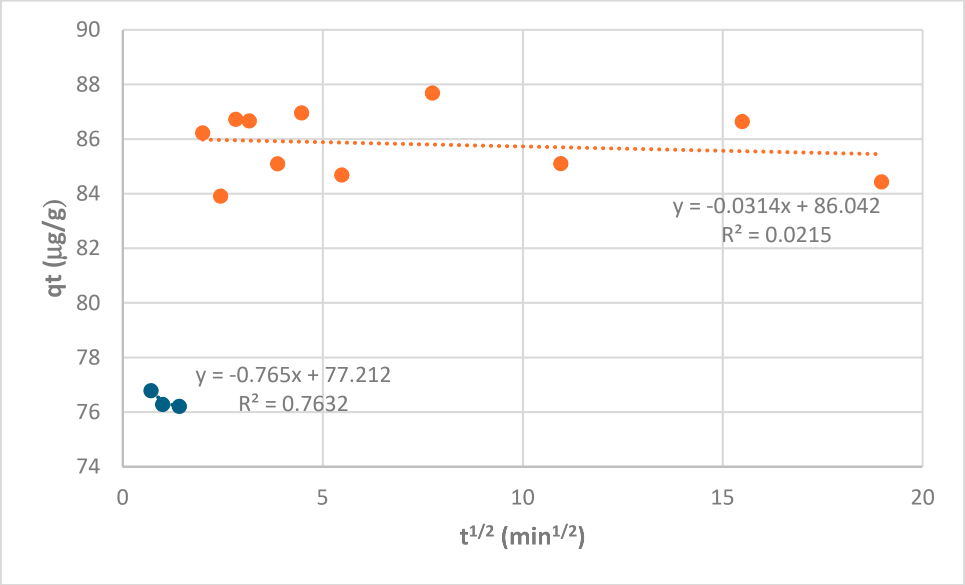

Figure S25. The WID kinetic model for the adsorption of KOL onto NaP1_C.

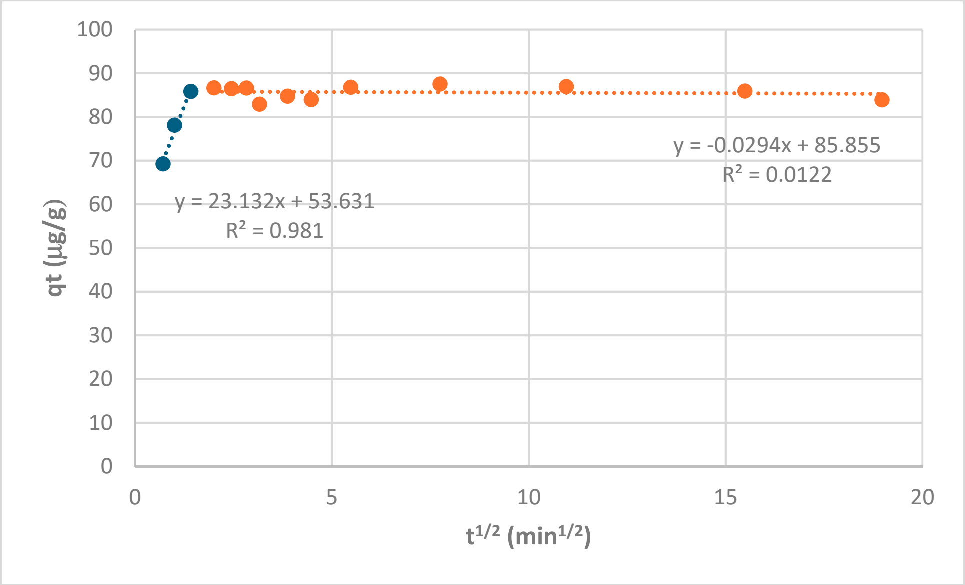

Figure S26. The WID kinetic model for the adsorption of KOL onto NaP1_FA.

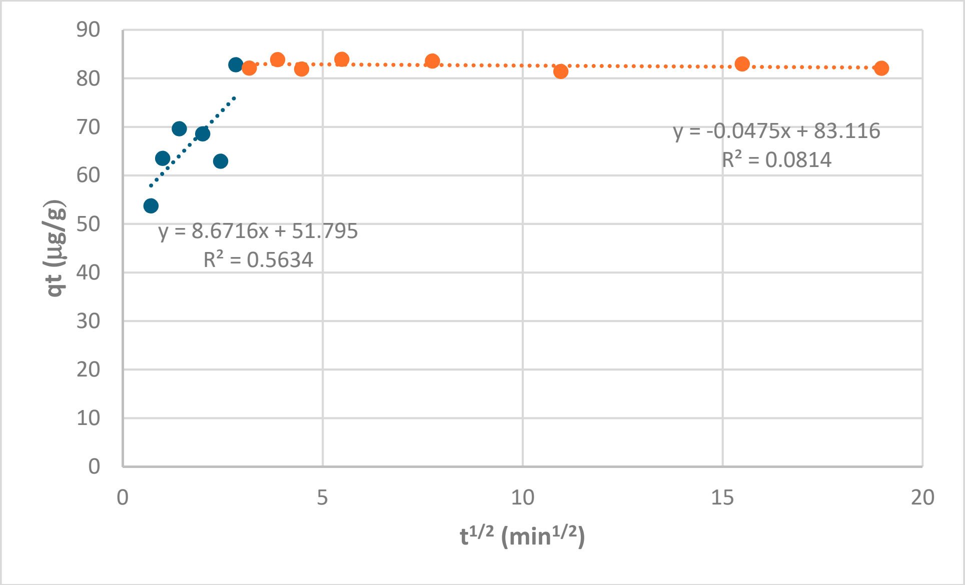

Figure S27. The WID kinetic model for the adsorption of FLUO onto NaP1_C.

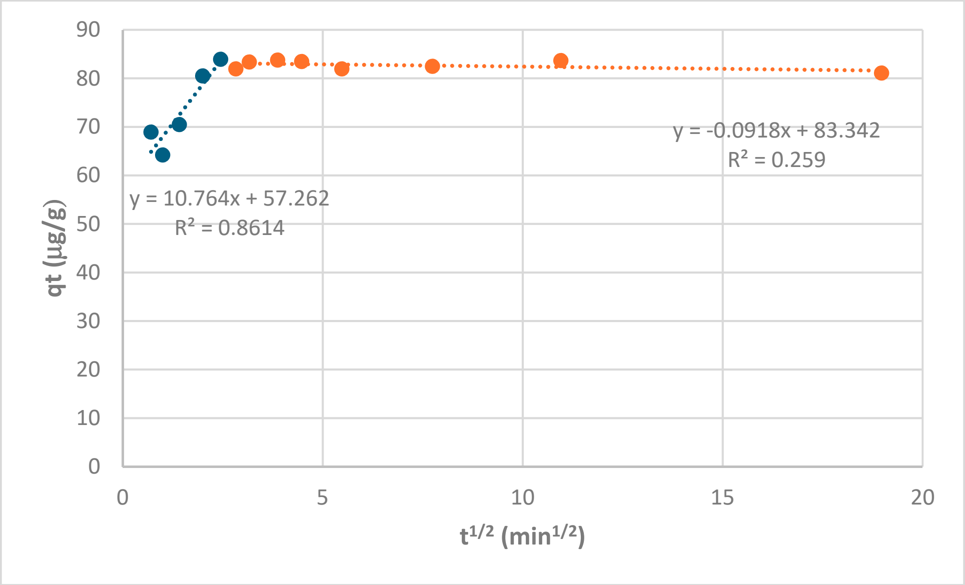

Figure S28. The WID kinetic model for the adsorption of FLUO onto NaP1_FA.

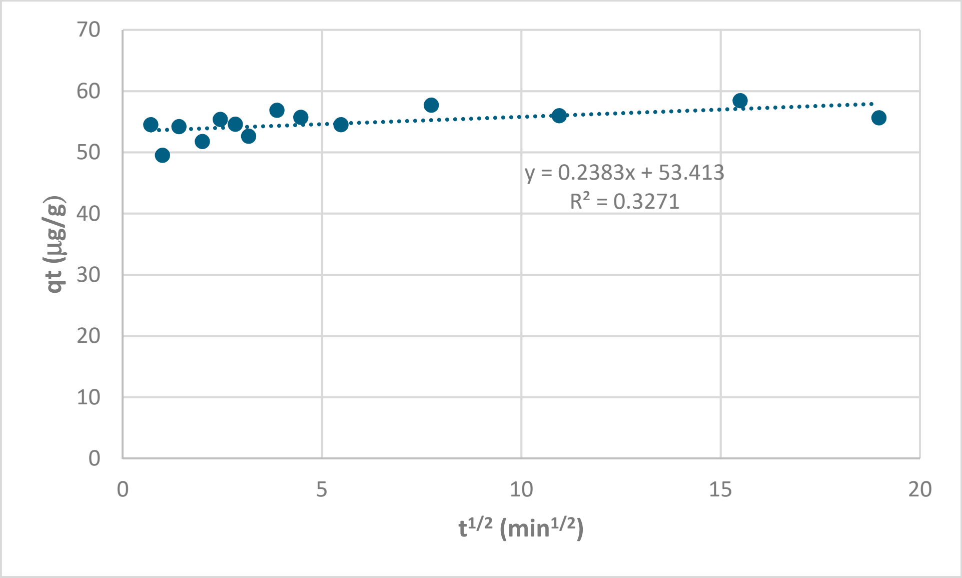

Figure S29. The WID kinetic model for the adsorption of AMO onto NaP1_C.

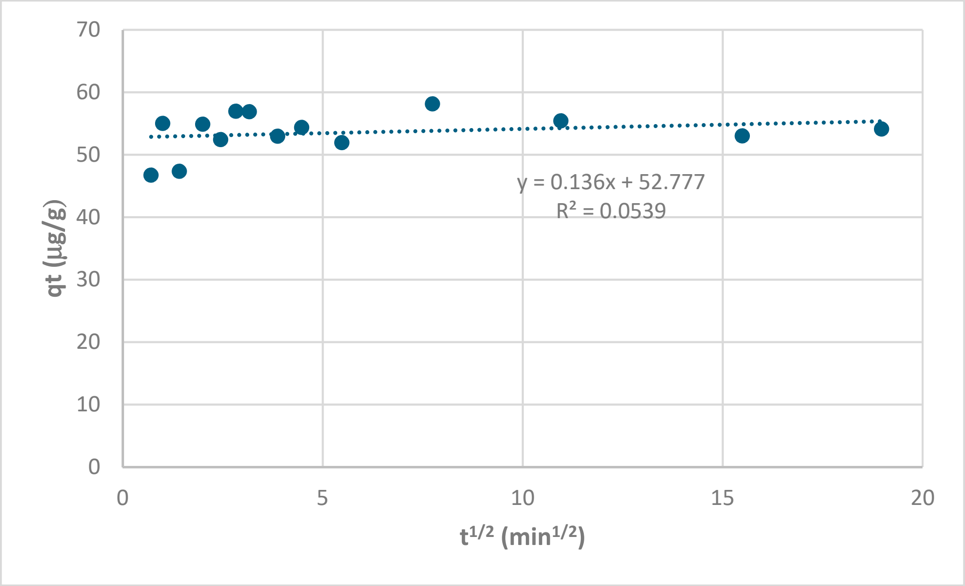

Figure S30. The WID kinetic model for the adsorption of AMO onto NaP1_FA.

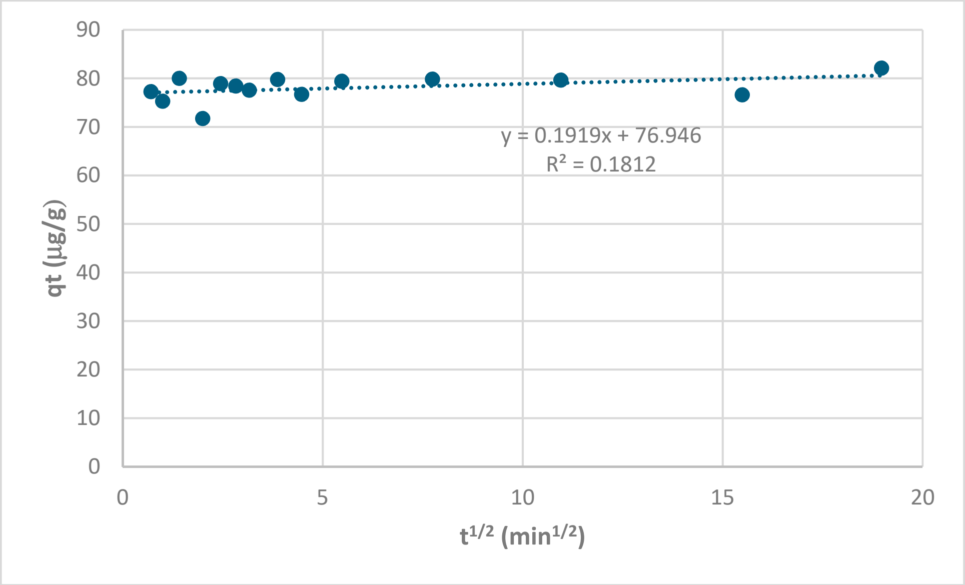

Figure S31. The WID kinetic model for the adsorption of EST onto NaP1_C.

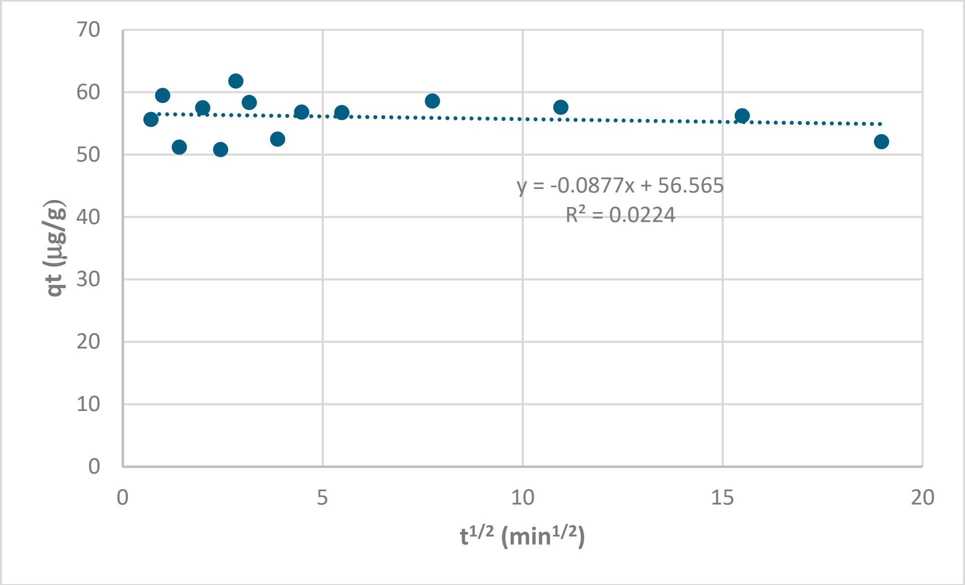

Figure S32. The WID kinetic model for the adsorption of EST onto NaP1_FA.

References

[1] Osmari, T.A.; Gallon, R.; Schwaab, M.; Barbosa-Coutinho, E.; Severo, J.B.; Pinto, J.C. Statistical Analysis of Linear and Non-Linear Regression for the Estimation of Adsorption Isotherm Parameters. *Adsorp Sci Technol* **2013**, *31(5)*, 433-458, doi: 10.1260/0263-6174.31.5.433.

[2] Ho, Y.S. Isotherms for the Sorption of Lead onto Peat: Comparison of Linear and Non-Linear Methods. *Polish J Environ Studies* **2006**, *15(1)*, 81-86.

[3] Mudhoo, A.; Pittman Jr., C.U. The Dubinin-Radushkevich models: Dissecting the p_s/p to c_s/c_e replacement in solid-aqueous interfacial adsorption and tracking the validity of E = 8 kJ mol^{-1} for assigning sorption type. *Design* **2023**, *198*, 370-402, doi: 10.1016/j.cherd.2023.09.020.
[4] Tran, H.N. Applying Linear Forms of Pseudo-Second-Order Kinetic Model for Feasibly Identifying Errors in the Initial Periods of Time-Dependent Adsorption Datasets. *Water* **2023**, *15(6)*, 1231, doi.org/10.3390/w15061231.
[5] Revellame, E.D.; Fortela, D.L.; Sharp, W.; Hernandez, R.; Zappi, M.E. Adsorption kinetic modeling using pseudo-first order and pseudo-second order rate laws: A review. *Clean Engineering Technol* **2020**, *1*, 100032, doi.org/10.1016/j.clet.2020.100032.
[6] Lian, Q.; Yao, L.; Ahmad, Z.U.; Gang, D.D.; Konggidinata, M.I.; Gallo, A.A.; Zappi, M.E. Enhanced Pb(II) adsorption onto functionalized ordered mesoporous carbon (OMC) from aqueous solutions: the important role of surface property and adsorption mechanism. *Environ Sci Pollut Res* **2020**, *27*, 23616-23630, https://doi.org/10.1007/s11356-020-08487-9.
